# Target preference of Type III-A CRISPR-Cas complexes at the transcription bubble

**DOI:** 10.1038/s41467-019-10780-2

**Published:** 2019-07-05

**Authors:** Tina Y. Liu, Jun-Jie Liu, Abhishek J. Aditham, Eva Nogales, Jennifer A. Doudna

**Affiliations:** 1 Department of Molecular and Cell Biology, University of California, Berkeley, CA 94720 USA; 2 California Institute for Quantitative Biosciences, Berkeley, CA 94720 USA; 3 Molecular Biophysics & Integrated Bioimaging Division, Lawrence Berkeley National Laboratory, Berkeley, CA 94720 USA; 4 Department of Chemical & Biomolecular Engineering, University of California, Berkeley, CA 94720 USA; 5 Howard Hughes Medical Institute, University of California, Berkeley, CA 94720 USA; 6 Gladstone Institutes, San Francisco, CA 94158 USA; 7 Innovative Genomics Institute, University of California, Berkeley, CA 94720 USA; 8 Department of Chemistry, University of California, Berkeley, CA 94720 USA

**Keywords:** Nucleases, RNA, CRISPR-Cas systems, Transcription, Electron microscopy

## Abstract

Type III-A CRISPR-Cas systems are prokaryotic RNA-guided adaptive immune systems that use a protein-RNA complex, Csm, for transcription-dependent immunity against foreign DNA. Csm can cleave RNA and single-stranded DNA (ssDNA), but whether it targets one or both nucleic acids during transcription elongation is unknown. Here, we show that binding of a *Thermus thermophilus (T*. *thermophilus*) Csm (TthCsm) to a nascent transcript in a transcription elongation complex (TEC) promotes tethering but not direct contact of TthCsm with RNA polymerase (RNAP). Biochemical experiments show that both TthCsm and *Staphylococcus epidermidis* (*S. epidermidis*) Csm (SepCsm) cleave RNA transcripts, but not ssDNA, at the transcription bubble. Taken together, these results suggest that Type III systems primarily target transcripts, instead of unwound ssDNA in TECs, for immunity against double-stranded DNA (dsDNA) phages and plasmids. This reveals similarities between Csm and eukaryotic RNA interference, which also uses RNA-guided RNA targeting to silence actively transcribed genes.

## Abstract (web summary)

Type III CRISPR-Cas systems are able to target transcriptionally active DNA sequences in phages and plasmids. Here, the authors reveal the mechanism of the target nucleic acid preference of Type III-A CRISPR-Cas complexes at the transcription bubble by a combination of structural and biochemical approaches.

## Introduction

CRISPR-Cas (clustered regularly interspaced short palindromic repeat-CRISPR associated) systems are prokaryotic adaptive immune systems that employ RNA-guided effector complexes to degrade foreign DNA or RNA^1,2^. Type III systems, which use multi-protein complexes containing a single CRISPR RNA (crRNA) molecule, are widespread in bacteria and archaea, and are possibly the most ancient type of CRISPR-Cas system^2,3^. Type III-A CRISPR-Cas loci, a major subtype, encode five proteins comprising the Csm targeting complex — Cas10/Csm1, Csm2–Csm5 — as well as the Csm6 ribonuclease^3^.

Type III-A systems can target DNA in vivo, and confer immunity against DNA phages or plasmids^4–6^. DNA targeting requires transcription across the target sequence to produce an RNA transcript that is complementary to the crRNA^4–6^. Csm complexes recognize and cleave complementary single-stranded RNA (ssRNA) oligonucleotides in vitro^6–8^. RNA cleavage requires the catalytic activity of Csm3^6,8,9^. Recognition of the target RNA also activates a nonspecific ssDNA cleavage activity in the Cas10/Csm1 subunit of Csm^9–13^. Transcription-dependent immunity has been proposed to involve recruitment of Csm to transcriptionally active DNA, but what exactly occurs following recruitment is not clear^6,9,10^. Csm has been proposed to target nascent RNA transcripts, ssDNA unwound by RNAP, or both substrates at the transcription bubble^5,9–13^. Whether interactions between Csm and RNAP are involved in targeting at the transcription bubble is not known. Although Csm complexes did not co-purify with RNAP when isolated from their native hosts, those purifications were conducted in the absence of an actively transcribed DNA target sequence in the cell^7,14^.

To determine what nucleic acid substrate Csm targets at the transcription bubble, we used single-particle electron microscopy (EM) and biochemical studies to investigate the interactions and activity of RNA-guided Csm complexes upon recruitment to reconstituted transcription elongation complexes (TECs). We show that binding to a nascent RNA transcript tethers TthCsm to the TEC but does not trigger a direct interaction with RNAP or ssDNA in the transcription bubble. Biochemical experiments with TthCsm and SepCsm show that they catalyze transcript degradation, but not cutting of the DNA at the transcription bubble. Taken together, these results suggest that Type III-mediated immunity involves RNA transcript degradation by Csm complexes rather than direct cleavage of DNA at the transcription bubble. The lack of a physical interaction between Csm and RNAP also helps to explain the prevalence of Type III CRISPR-Cas systems across different species of bacteria and archaea, as there would be no requirement for adaptation to specific RNAPs in each new host. These findings reveal similarities between Type III CRISPR-Cas systems and the eukaryotic RNA interference pathway, which uses an RNA-guided complex, RISC (RNA-induced silencing complex), to cleave RNA transcripts for gene silencing^15^.

## Results

### RNA-mediated tethering of TthCsm to the transcription bubble

**Fig. 1:**
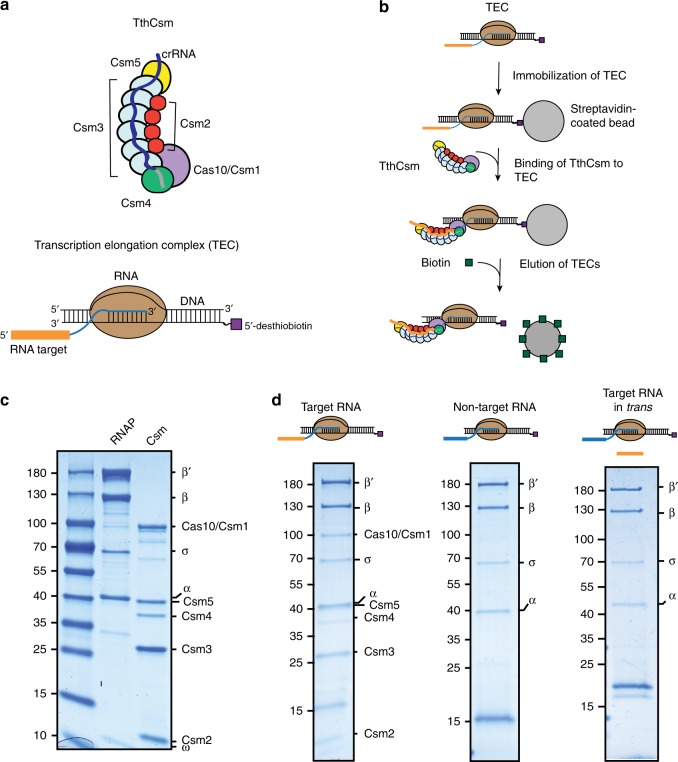
Tethering of TthCsm to a transcription elongation complex (TEC) by a complementary nascent RNA transcript. **a** Schematic of the TthCsm complex and the TEC. TthCsm subunits are labeled. RNAP; RNA polymerase. The sequence in the RNA transcript that is complementary to the crRNA in TthCsm is highlighted in orange. A 5´-desthiobiotin moiety on the template strand of DNA in the TEC is labeled. **b** Schematic of the pull-down assay. The TEC was immobilized on streptavidin beads using a desthiobiotin group on the DNA. TthCsm was incubated with the TEC, after which the beads were washed to remove unbound complexes, and biotin was added to elute the TECs from the beads. **c** Sodium dodecyl sulfate polyacrylamide gel electrophoresis (SDS-PAGE) of the purified *T. thermophilus* RNAP (α, β´, β, σ, ϖ) and TthCsm (Cas10/Csm1, Csm2-Csm5) complexes. A molecular weight marker was run for comparison in the leftmost lane and sizes are indicated in kilodaltons. **d** Pull-down assays were done with TECs containing a target (left), non-target (middle), or target RNA transcript added in *trans* (right). The elutions were analyzed by SDS-PAGE. The presence of RNAP and TthCsm subunits is indicated. The positions of the molecular weight markers in (**c**) are shown. Uncropped gel images for (**c**, **d**) are available online in the Source Data file

The TthCsm complex comprises a crRNA bound by a single Cas10/Csm1 enzyme together with multiple Csm2 and Csm3 subunits and two capping subunits, Csm4 and Csm5 (Fig. 1a)^9^. We previously reconstituted the TthCsm and showed that binding to a crRNA-complementary target RNA triggers TthCsm to cleave both the bound RNA and nonspecific ssDNA sequences^9^. To test whether TthCsm interacts with RNA and DNA at an RNAP-bound transcription bubble, we first tested whether nascent RNA recruits TthCsm to a transcription bubble in vitro. Purified *T*. *thermophilus* RNAP was assembled with an R-loop containing a DNA mismatch bubble and an RNA transcript; the transcript bears a template DNA-complementary 3´ sequence and a crRNA-complementary 5´ target sequence (Fig. 1a). These reconstituted TECs were immobilized on streptavidin beads, incubated with TthCsm under conditions that foster binding but not catalysis, washed to remove unbound TthCsm, and eluted (Fig. 1b). We observed that TthCsm subunits co-eluted with RNAP only when the RNA contained a sequence that was complementary to the crRNA (Fig. 1c, d, “target RNA”), but not when the target was replaced by an unrelated sequence (Fig. 1c, d, “non-target RNA”). Decreasing the length of the RNA between the target sequence and the DNA bubble from 32 nucleotides (nts) to 11 nts also decreased the pull-down efficiency, likely because the target sequence in the shortest RNA was not extended enough beyond the surface of the RNAP to allow TthCsm to bind it (Supplementary Fig. 1). Taken together, these data show that recognition of complementary RNA is required to recruit Csm to the TEC.

To test whether the interaction between TthCsm and the TEC is due to transcript tethering or to an RNA-induced conformational change in TthCsm that leads to a direct interaction with RNAP, we performed a TEC pull-down experiment in which TthCsm was supplied with a complementary target RNA in *trans*, i.e. as a free oligonucleotide that was not linked to the RNAP (Fig. 1c, d, target RNA in *trans*). This did not result in co-elution of TthCsm with the RNAP subunits, indicating that the RNA transcript acts as a tether between TthCsm and RNAP.

**Fig. 2:**
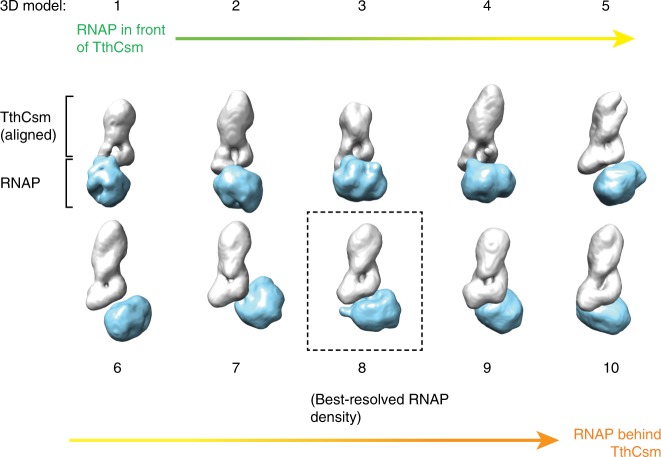
Visualization of TthCsm tethered at the TEC. Negative-stain 3D EM reconstructions of the TthCsm-TEC complex are shown. TthCsm is colored gray, and the RNAP is colored light blue. The 3D models are numbered from 1-10. The orientation of TthCsm is aligned in all of the 3D models and a colored gradient arrow is used to illustrate how RNAP changes its orientation from in front to behind TthCsm. The boxed model 8, which has the most well-resolved features on the RNAP, was used for structural modeling in Fig. 3

To visualize the TthCsm at the transcription bubble, co-eluted TthCsm-TEC complexes were analyzed by negative stain electron microscopy (EM). The complex appeared as two types of particles – an elongated particle that represents TthCsm and a more compact, circular particle that is consistent with structures of elongating *T. thermophilus* RNAP (Supplementary Fig. 2a,b)^7,16^. We picked particles that contained both TthCsm and RNAP, and performed 2D and 3D classification, revealing several distinct 3D models for the TthCsm-TEC complex (Fig. 2 and Supplementary Fig. 2a,b). The 2D class averages and 3D reconstructions showed that TthCsm adopts different positions relative to the RNAP, consistent with loose, transcript-based tethering between RNAP and TthCsm (Fig. 2 and Supplementary Fig. 2a,b). When we aligned the TthCsm densities in all of the 3D reconstructions, the RNAP appears to swing from in front to behind TthCsm (Fig. 2). Particles were also evenly distributed across the different classes, indicating that there was no preferred “docked” state of TthCsm with RNAP.

To ensure that the dynamics between TthCsm and RNAP were not caused by disruption of TthCsm-RNAP contacts during the negative-staining procedure, we also used cryo-EM to visualize these complexes (Supplementary Fig. 2c, d). Particle picking and 2D classification of the cryo-EM data revealed a similar orientation and degree of mobility of TthCsm around the RNAP as the negative stain EM results (Fig. 2 and Supplementary Fig. 2d). This suggests that RNA binding brings TthCsm close to the TEC but does not induce a stable interaction with the RNAP. Instead, the complementary nascent RNA acts as a flexible linker between the complexes. Taken together with the pull-down results, this shows RNA-guided recognition of the nascent transcript tethers TthCsm near the TEC but does not induce a direct interaction with the RNAP.

### Substrate positioning in the TthCsm-TEC complex

**Fig. 3:**
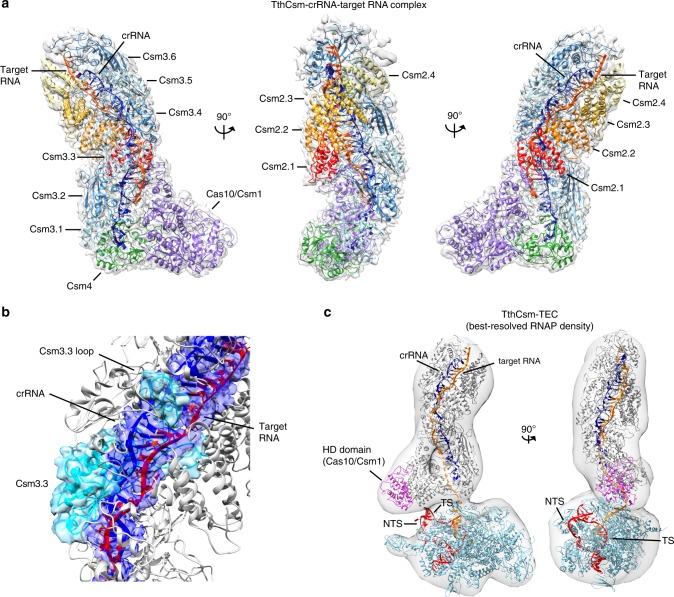
Cryo-EM structures of target RNA-bound TthCsm and modeling of the TthCsm-TEC complex. **a** Cryo-EM structure and model of target RNA-bound TthCsm. TthCsm subunits, crRNA, and target RNA are labeled in the different views. Each view is rotated by 90˚, as indicated by arrows. **b** Detailed view of the discontinuous crRNA and target RNA hybrid, showing the base pairs fit into the EM density of the RNA hybrid. A loop in each Csm3 subunit inserts after every 5-nt segment and flips out the 6th base. The EM density of the crRNA:target RNA hybrid and the modeled crRNA strand are colored dark blue, and the modeled target RNA strand is colored red-orange. The EM density and model corresponding to the third Csm3 subunit (Csm3.3) is colored light blue. **c** Models of RNA-bound TthCsm and the TEC complex fit into the negative stain EM map. The HD domain of Cas10/Csm1 is colored pink, the nontemplate strand (NTS) and template strand (TS) are colored red, the RNA transcript is colored orange, and the crRNA is colored dark blue. A dotted orange line is used to represent the segment of RNA between the RNA exit channel and the TthCsm bound target sequence. TthCsm is colored gray and the protein subunits of the RNAP are colored light blue. The orientation of the RNAP was determined using the features of the EM density map (3D model 8 in Fig. 2)

To determine how TthCsm recognizes RNA and ssDNA substrates, we used cryo-EM to analyze TthCsm bound to a complementary target RNA and a noncomplementary ssDNA molecule in the presence of EDTA (ethylenediaminetetraacetic acid) to chelate magnesium ions and thereby inhibit nucleic acid cleavage (Supplementary Fig. 3a). Electrophoretic mobility shift experiments (EMSAs) showed that a small, fixed amount of radiolabeled ssDNA was not fully bound by TthCsm at concentrations up to 10 µM (Supplementary Fig. 3b**)**, indicating TthCsm has a weak affinity for ssDNA. A 3D reconstruction of the TthCsm sample (3.8 Å resolution), which contained both target RNA and noncomplementary ssDNA, revealed a complex comprising one copy each of Cas10/Csm1 and Csm4, six Csm3 subunits, and four Csm2 subunits bound to a crRNA:target RNA hybrid (Fig. 3a and Supplementary Figs. 3c,d, 4, and 5). Though we added an excess of ssDNA to the TthCsm sample, we did not observe density corresponding to ssDNA in the DNase subunit, Cas10/Csm1, consistent with the low affinity of TthCsm for ssDNA (Supplementary Fig. 3b**)**. This subunit stoichiometry is similar to that of a TthCsm complex determined previously by native mass spectrometry, with the exception of a missing Csm5 subunit^9^. The additional density at the top of the TthCsm complex likely corresponds to Csm5 but could not be modeled due to poorly resolved density (Fig. 3a). During 3D classification, a shorter complex was also observed, which likely corresponds to a second complex containing four Csm3 and three Csm2 subunits, as detected previously by native mass spectrometry (Supplementary Fig. 4a, 3D class with 29.1% of particles)^9^. The larger TthCsm complex has an i:i + 2 ratio of Csm2 to Csm3 subunits that differs from the i:i + 1 ratio of Type III-A Csm complexes from *Streptococcus thermophilus* (*S. thermophilus*) and *Thermococcus onnurineus* (*T. onnurineus*), revealing a unique complex stoichiometry (Fig. 3a and Supplementary Fig. 5a)^17,18^.

TthCsm recognizes target RNA by base-pairing with the crRNA in discontinuous 5-nt segments, with the lowest segment flanked by the C-terminal domain of Cas10/Csm1 and Csm3, and each subsequent segment flanked by Csm2 and Csm3 subunits (Fig. 3a and Supplementary Fig. 5a). A loop from Csm3 flips out every 6^th^ nucleotide in the target RNA to position each site for cleavage (Fig. 3a and Supplementary Fig. 5a). The presence of six Csm3 subunits, the catalytic component responsible for RNA cleavage, also explains TthCsm’s ability to recognize and cut target RNA at six different sites^7,9^.

The histidine-aspartate (HD) nuclease domain of Cas10/Csm1, positioned at the “toe” of the TthCsm, is responsible for ssDNA cleavage, and has been proposed to directly contact and cleave the nontemplate DNA at the transcription bubble upon transcript recognition^9–12^. To determine the positioning of DNA and RNA relative to TthCsm, we modeled the negative-stain 3D density map containing the best-resolved RNAP (Fig. 2, “3D model 8”) with the pseudoatomic model of TthCsm and a complete *Thermus* transcription complex (Fig. 3c)^16,19^. In this model, TthCsm is positioned directly outside of the RNA exit channel, where it could bind the emerging transcript (Fig. 3c). This model also suggests that the path of the unwound nontemplate DNA strand would run along the opposite side of the RNAP from TthCsm (Fig. 3c). In this orientation, it would be difficult for TthCsm to access the unpaired region of the nontemplate DNA in the transcription bubble. Thus, this suggests a preference of TthCsm for binding the nascent RNA transcript, rather than unwound DNA at the transcription bubble.

### DNA and RNA cleavage by TthCsm at the TEC

**Fig. 4:**
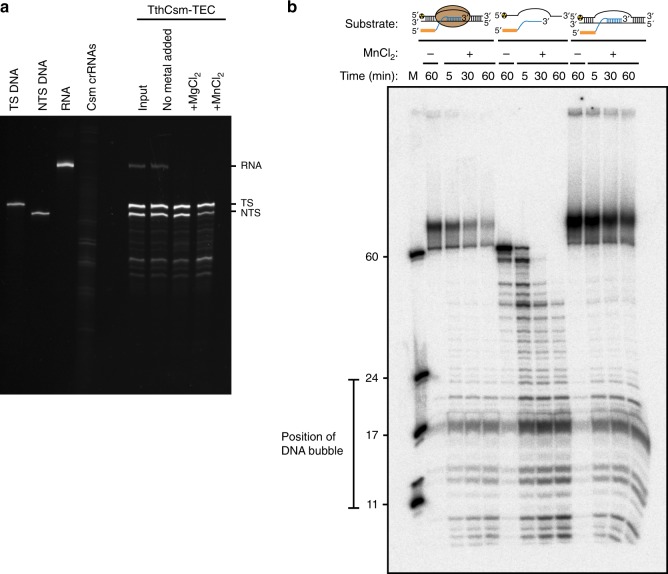
Target preference of TthCsm at the transcription bubble. **a** Cleavage of nucleic acids by TthCsm in the TthCsm-TEC complex was initiated by addition of MgCl_2_ or MnCl_2_ and reactions were incubated for 30 min at 65 ˚C. Products were analyzed by denaturing PAGE and SYBR gold staining. The nontemplate strand (NTS DNA), template strand (TS DNA), RNA transcript (RNA), and crRNAs contained in the TthCsm (Csm crRNAs) were loaded individually on the left half of gel for comparison. **b** TthCsm-catalyzed cleavage of the 5´-radiolabeled NTS. The substrates tested were the eluted TthCsm-TEC sample from the pull-down (left), the NTS DNA alone with the target RNA transcript, which includes a sequence complementary to the TthCsm crRNA (center), and an R-loop bubble composed of NTS DNA, TS DNA, and the target RNA transcript (right). A ssDNA marker was run in the leftmost lane (M), and the position of the unpaired region is shown. Uncropped gel images for (**a**, **b**) are available online in the Source Data file

We next tested whether the TthCsm cleaves DNA and RNA at the transcription bubble. The HD domain of Cas10/Csm1 catalyzes ssDNA cleavage, whereas the multiple Csm3 subunits catalyze RNA cleavage at 6-nt intervals along the RNA target^9^. Divalent metal ions were added to the co-eluted TthCsm-TEC complex after assembly and elution to initiate nucleic acid cleavage. TthCsm-catalyzed RNA cleavage occurs either in the presence of Mg^2+^ or Mn^2+^, but robust DNA cleavage only occurs in the presence of Mn^2+^ ions^7,9^. When MgCl_2_ was added, we observed disappearance of the band corresponding to RNA, but not the DNA (Fig. 4a). To determine if Csm also cleaved ssDNA at the mismatch bubble in the DNA, we added MnCl_2_. This led to complete degradation of RNA and partial degradation of the nontemplate strand of DNA (Fig. 4a). To determine if cleavage specifically occurred at the unpaired region of the DNA within the transcription bubble, we radiolabeled the nontemplate strand and analyzed the cleavage products by denaturing polyacrylamide gel electrophoresis (PAGE). In the TEC sample, TthCsm only cleaved DNA in the unpaired region and the 11-nt duplex upstream of this region (Fig. 4b). Transient unwinding of the short, 11-nt duplex at the assay temperature of 65 ˚C likely explains the cleavage in the duplex region, as the predicted melting temperature (T_m_) of this short sequence is ~46 ˚C. Testing of a substrate containing a 30-nt duplex (T_m_ of ~76 ˚C) upstream of the bubble showed dramatically reduced cleavage outside of the unpaired region (Supplementary Fig. 6).

While TthCsm can cleave unpaired ssDNA, it is not clear if the cleaved ssDNA is part of the transcription bubble, or instead is present in excess R-loops that are not bound by RNAP. Nuclease protection and structural data indicate that the unwound DNA in the transcription bubble is minimally exposed, with only the upstream half of the transcription bubble accessible to solvent^19–21^. Thus, if DNA cleavage occurs at the transcription bubble, the downstream half of the transcription bubble should be protected when compared to cleavage at an R-loop lacking RNAP. We found that the cleavage pattern of the unbound R-loop DNA was similar to that of the DNA in which RNAP was present (Fig. 4b), suggesting that DNA cleavage by the TthCsm occurs only on free R-loops lacking bound RNAP. Taken together, these results indicate that TthCsm cleaves RNA in the TEC, but cannot access the RNAP-bound ssDNA.

### Co-transcriptional targeting preference of SepCsm

**Fig. 5:**
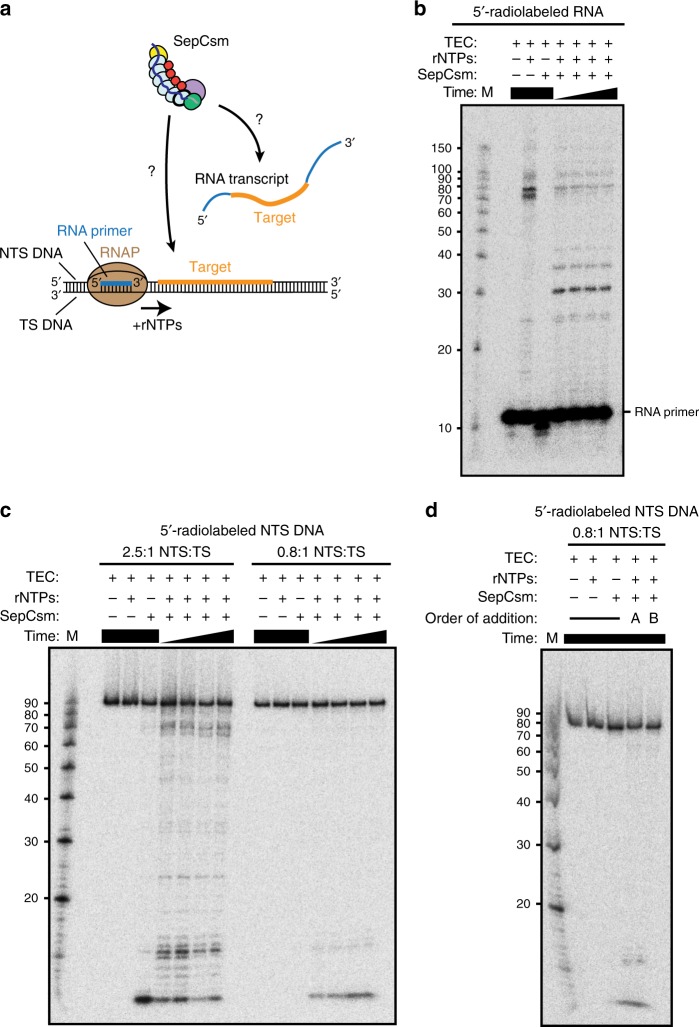
Testing co-transcriptional DNA and RNA targeting activities of SepCsm. **a** Schematic of the TEC used in the co-transcriptional DNA cleavage assay. The TEC was assembled with RNAP, a short RNA primer, nontemplate strand (NTS DNA), and template strand (TS DNA) of DNA. Ribonucleotides (rNTPs) were added to initiate transcription across the DNA and produce an RNA transcript. The NTS DNA and corresponding RNA transcript contains a target sequence (highlighted in orange) that is complementary to the crRNA guide sequence in SepCsm. **b** Co-transcriptional cleavage of 5´-radiolabeled RNA by SepCsm, analyzed by denaturing PAGE. Transcription was first initiated across the target sequence in the DNA by addition of rNTPs, followed by addition of SepCsm after 10 min. For reactions with both SepCsm and rNTPs added, samples were taken at 30, 60, 90, and 120 min after SepCsm addition. For all other reactions, samples were taken at 120 min. An RNA Decade™ marker (M) is loaded in the leftmost lane. **c** Co-transcriptional cleavage of 5´-radiolabeled NTS DNA by SepCsm, analyzed by denaturing PAGE. The assay was performed as in (**b**). The molar ratio of NTS to TS used in TEC assembly is given above the gel. A ssDNA marker (M) was loaded in the leftmost lane for comparison. **d** Comparison of co-transcriptional DNA cleavage by SepCsm when it is added before or after transcription is initiated. SepCsm was added either 10 min after (A) or 10 min before (B) rNTPs were added to initiate transcription of TECs containing a limiting amount of nontemplate strand DNA. After 30 min, reactions were halted and samples were analyzed as in **c**. All other reactions were incubated for the total length of the assay (40 min) before analysis. A ssDNA marker (M) was loaded in the leftmost lane for comparison. Uncropped gel images for (**b**–**d**) are available online in the Source Data file

Our results so far show that TthCsm cleaves nascent RNA transcripts, but not DNA at the transcription bubble. To determine if this preference is unique to TthCsm or a trait that is also found in other Type III-A complexes, we investigated the substrate preference of the Type III-A Csm effector from *S. epidermidis* (SepCsm). To test if SepCsm recognizes RNA transcripts, we purified SepCsm (Supplementary Fig. 7a,b**)** and performed co-transcriptional RNA cleavage experiments in which the RNA in the TEC was 5´-radiolabeled for detection, according to previously described procedures (Fig. 5a, b)^6^. Previously, transcript cleavage by SepCsm could not be detected in co-transcriptional assays due to the high amount of truncated transcripts produced by RNAP^6^. In our assays, we observed synthesis of the full-length RNA transcript by RNAP in the presence of ribonucleotides alone, without a significant amount of truncated transcripts (Fig. 5b). Cleavage of the full-length transcript at regular ~6-nt intervals occurred upon addition of SepCsm, consistent with Csm3-catalyzed ssRNA cleavage (Fig. 5b)^6^. This shows that SepCsm targets complementary RNA transcripts.

To test if SepCsm targets DNA in transcription bubbles, we used the same assay, but 5´-radiolabeled the nontemplate strand of DNA^6^. A previous study showed that SepCsm could cleave the nontemplate strand of DNA in a transcription-dependent manner, when its sequence was complementary to the crRNA^6^. However, in those experiments, the use of a 2.5-fold molar excess of nontemplate over template strand DNA could have complicated data interpretation due to cleavage of free, excess nontemplate ssDNA^6^. In addition, truncation of the gel images prevented detection of any smaller DNA fragments that could have resulted from SepCsm-catalyzed DNA cleavage in the absence of nucleotides^6^. To determine if any cleavage occurred in the absence of transcription, we first performed co-transcriptional DNA cleavage experiments using the original 2.5:1 molar ratio of nontemplate to template strand, but reduced the gel running time so that smaller cleavage products could be visualized (Fig. 5a, c)^6^. Addition of ribonucleotides alone did not stimulate nontemplate DNA cleavage, but surprisingly, addition of SepCsm in either the presence or absence of ribonucleotides led to a reduction in the amount of full-length DNA and the appearance of cleavage products (Fig. 5c and Supplementary Fig. 7c, d). In the absence of ribonucleotides, the majority of products accumulated at sizes < 20 nts, with only a small amount of ~70-nt (nucleotide) cleavage fragments remaining (Fig. 5c**)**. In conditions with ribonucleotides added, there were more fragments at ~70 nts (Fig. 5c and Supplementary Fig. 7d**)**. We also ran the samples for a longer period of time on the gel to show that, without retention of the smaller products on the gel, we observed a similar pattern of cleavage as reported by Marraffini and colleagues (Supplementary Fig. 7c)^6^. However, our data now show that degradation of the nontemplate strand of DNA in fact occurs in the absence of transcription, consistent with cleavage occurring on excess nontemplate ssDNA.

To determine whether SepCsm-dependent DNA cleavage was occurring on transcription bubbles or excess ssDNA, we reduced the ratio of nontemplate to template strand DNA from 2.5:1 to 0.8:1 (Fig. 5c and Supplementary Fig. 7c). This resulted in a dramatic reduction in the amount of cleavage products, suggesting that the observed cleavage was occurring primarily on excess, free nontemplate DNA (Fig. 5c and Supplementary Fig. 7c). To exclude that differences in DNA cleavage were due to a transcriptional defect of TECs assembled with limiting nontemplate strand DNA, we incorporated a 5´-radiolabeled RNA primer into the TECs to detect synthesis of full-length transcripts. This showed a similar level of full-length RNA transcripts synthesized by TECs containing either a 2.5:1 or 0.8:1 ratio of nontemplate to template strand (Supplementary Fig. 8). Also, in previous experiments, SepCsm was added 10 minutes after ribonucleotide addition^6^, but transcription by RNAP plateaus ~5 min after ribonucleotide addition (Supplementary Fig. 8). This occurs because only a single round of transcription is possible using these DNA templates. To determine if the reduced DNA cleavage at the 0.8:1 ratio was due to a lack of active transcription, we compared DNA cleavage when SepCsm was added before vs. after ribonucleotide addition (Fig. 5d). The amount of cleavage products observed when using either order of addition was identical, indicating that no DNA cleavage occurred while the RNAP was actively transcribing (Fig. 5d**)**. The small amount of DNA cleavage products in the experiments with limiting nontemplate DNA may instead be explained by to exposure of ssDNA in R-loops without RNAP bound^22^. These sometimes form as a by-product of transcription, when the transcribed RNA anneals to the complementary template DNA strand and keeps the DNA duplex unpaired^22^. In summary, these experiments show that SepCsm is similar to TthCsm in that it cleaves RNA transcripts, but not ssDNA at transcription bubbles.

## Discussion

A distinguishing feature of Type III CRISPR-Cas systems is their ability to target transcriptionally active DNA sequences in phages and plasmids^5,6,23^. It has been proposed that the Type III-A Csm complex localizes to the transcription bubble, where DNA is transiently unwound by RNAP as it synthesizes RNA^5,6,9,10^. Csm has intrinsic RNA and ssDNA cleavage activities, and thus could cleave either nascent transcripts, unpaired DNA at the transcription bubble, or both^6–10,24^. Whether it interacts with the RNAP during targeting is unknown. Using reconstituted TECs, we found that recognition of a target sequence in a nascent transcript can recruit TthCsm to a TEC, but does not trigger a stable interaction of TthCsm with the RNAP or the transcription bubble (Figs. 1, 2). The nascent RNA acts as a tether between TthCsm and TEC; as the RNAP transcribes, the distance between TthCsm and the TEC would increase. TthCsm could also bind and cleave fully synthesized, free RNA transcripts without being recruited to transcriptionally active DNA. This suggests that localization of TthCsm to transcription bubbles is solely RNA-mediated and would likely be transient. RNA cleavage and dissociation of the fragments would also contribute to the eventual release of Csm complexes from the TEC^10,11^. The lack of an interaction between TthCsm and RNAP suggests that direct cleavage of the unwound DNA bubble by Csm during transcription would be difficult. However, this trait may explain how Type III CRISPR-Cas systems became widespread across different bacterial and archaeal hosts, as no specific interface on the host RNAP would need to be recognized for its function.

We also elucidated the structural basis of RNA target positioning by TthCsm. The cryo-EM structure of TthCsm revealed that target RNA is recognized by base-pairing with the crRNA in discontinuous 5-nt segments, with every 6^th^ nucleotide flipped out by a loop from Csm3 (Fig. 3 and Supplementary Fig. 5a). The role of Csm3 is similar to that of the Cmr4 subunit of Type III-B Cmr and the Cas7 subunit of Type I Cascade complexes, supporting the idea of a common evolutionary ancestor for Class 1 effectors^25–28^. While the overall architecture of the TthCsm is similar to that of Type III-A Csm complexes from *T. onnurineus* and *S. thermophilus* (TonCsm; SthCsm), TthCsm contains additional Csm3 and Csm2 subunits that allows it to accommodate a longer crRNA (Fig. 3a and Supplementary Fig. 5a). TthCsm can position 24 nts of target RNA for cleavage, instead of ~18 nts in TonCsm and SthCsm (Fig. 3a and Supplementary Fig. 5a**)**^17,18^. Also, while TonCsm and SthCsm typically have one more copy of Csm3 than Csm2, TthCsm has two more Csm3 than Csm2 subunits. This suggests that the ratio of Csm3:Csm2 subunits in the complex may be different for larger Type III assemblies^17,18^. Larger Type III interference complexes may provide enhanced immunity, similar to Type I Cascade complexes containing extended crRNA guides^29^.

We also showed that TthCsm cleaves nascent transcripts, but does not interact with or cleave the DNA in the transcription bubble (Figs. 3 and 4). Docking of a pseudoatomic model of target RNA-bound TthCsm and a composite model of the *Thermus* TEC with a complete DNA bubble into the negative stain EM map showed that the TthCsm was positioned outside the RNA exit channel, where it would bind the nascent transcript as it emerges from the RNAP. Pull-down assays also indicate that the entire target sequence must be synthesized and extended beyond the surface of the RNAP for TthCsm to bind it (Supplementary Fig. 1). When we tested the activity of TthCsm in the co-eluted TthCsm-TEC complex, we found that TthCsm catalyzed cleavage of the nascent RNA transcript as well as free R-loop DNA, but not DNA within the transcription bubble (Fig. 4). Since TthCsm could still cleave ssDNA in free R-loops, the lack of cleavage at the transcription bubble is most likely due to inaccessibility of the ssDNA to the HD nuclease domain of Cas10/Csm1. Only about 5-6 nts of the nontemplate strand DNA would be exposed on the surface of the RNAP, based on structural and nuclease protection studies on transcription complexes^19–21^. The nontemplate strand of DNA is positioned on the opposite side of the RNAP relative to TthCsm in our structural model (Fig. 3c). Lastly, the affinity of TthCsm for ssDNA is also significantly lower than its affinity for complementary target RNA (Supplementary Fig. 3b)^9^. Thus, silencing of transcriptionally active DNA by TthCsm most likely relies on recognition and cleavage of RNA transcripts, rather than cleavage of ssDNA that is transiently unwound by RNAP.

SepCsm is the only Type III complex for which co-transcriptional DNA and RNA targeting has been reconstituted in vitro and these experiments have been cited in support of a model in which DNA cleavage occurs at transcription bubble^6,10–12,30,31^. However, clear interpretation of the results in the previous study is difficult, given that excess, free nontemplate ssDNA was present in the co-transcriptional DNA cleavage experiments. Here, using a limiting concentration of nontemplate DNA, we found that SepCsm cleaves complementary RNA transcripts, but not ssDNA unwound by RNAP in transcription bubbles (Fig. 5). Like TthCsm, SepCsm still exhibits activity towards free ssDNA, indicating that the lack of cleavage at the transcription bubble is likely due to insufficient exposure or accessibility of ssDNA. Thus, our data show that Csm complexes from both thermophilic and mesophilic bacteria exhibit a targeting preference for RNA transcripts instead of ssDNA in TECs.

**Fig. 6:**
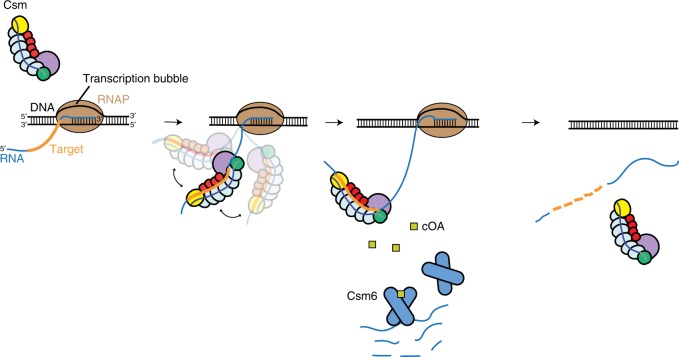
Model for transcription-dependent immunity by RNA-guided Type III CRISPR-Cas systems. Transcription across a DNA target generates a nascent transcript that is complementary to the crRNA of Csm (complementary target sequence in RNA is colored orange). Binding to the transcript flexibly tethers TthCsm to the TEC, but does not lead to an interaction of TthCsm with the RNAP or the DNA in the transcription bubble. Csm cleaves transcripts using its Csm3 subunit, instead of cleaving the ssDNA at the transcription bubble. While bound to RNA, Csm also synthesizes cyclic oligoadenylate (cOA) molecules, which binds to and activates the Csm6 RNase for transcript degradation

This leads to the question of how Type III systems defend against transcriptionally active DNA. Our in vitro data suggest that TthCsm could target R-loops (Fig. 4), which form when an RNA transcript invades the DNA duplex^22^. However, it is unclear whether R-loops would persist long enough for Type III effectors to target them in vivo, as R-loops are resolved in the cell by RNase H, a nuclease that specifically cleaves the RNA in DNA:RNA hybrids^22^. A more plausible explanation is that the Type III system primarily depends on its RNase activity for immunity against dsDNA invaders. In addition to the Csm3 RNase in the effector complex, Type III-A CRISPR-Cas loci also encode a nonspecific RNase, Csm6^2^. During immunity, recognition of a complementary transcript not only stimulates target RNA cleavage by Csm3, but also activates synthesis of a cyclic oligoadenylate molecule (cOA) by the Cas10/Csm1 palm polymerase domain (Fig. 6)^24,32^. Binding of the cOA to Csm6 stimulates its RNase activity, which results in degradation of host and invader transcripts (Fig. 6)^24,32,33^. Several in vivo studies support this revised view of transcription-dependent immunity. First of all, mutation of the catalytic GGDD motif in Cas10’s palm domain or deletion of Csm6 abolished anti-plasmid immunity, while mutation of the catalytic HD motif in Cas10, which is required for ssDNA cleavage, did not^13,34,35^. Csm6 is also required for DNA clearance when transcription across a plasmid target is infrequent^33^. Inactivation of Csm3 and Csm6 also prevented immunity against late-expressed targets in dsDNA phages^36^. Taken together with our results, this suggests that RNA recognition and cleavage by Csm and Csm6, rather than ssDNA cleavage, is the preferred mode of Type III immunity against dsDNA invaders. RNA degradation by Type III CRISPR-Cas systems could silence genes that are required for plasmid or phage replication, which would lead to loss of DNA without direct DNA cleavage by Csm or stall progression of the infection until Csm has an opportunity to access invader ssDNA^33,34,36^. Nonspecific transcript degradation by Csm6 could also prevent spread of a phage or plasmid infection in a bacterial population by inducing cell death or quiescence of infected cells^24,32,33^. Further studies will be needed to elucidate the details of how exactly RNA degradation by Csm6 contributes to transcription-dependent immunity against DNA invaders.

In summary, our study defines the preferred target of Csm at the transcription bubble as RNA transcripts, rather than unwound ssDNA. Our results suggest that recognition and cleavage of transcripts by Csm is the primary mode of transcription-dependent immunity against foreign DNA (Fig. 6). This reveals parallels between Type III systems and eukaryotic RNA interference, which also uses an RNA-guided complex, RISC, to silence specific genes by degradation of their associated transcripts^15^. Our study also raises new questions about the function of the ssDNase activity in Type III complexes. Since Csm does not target transcription bubbles, we speculate that it could target other sources of exposed ssDNA in the cell, such as DNA replication intermediates^37^ and R-loops^22^, or provide immunity against ssDNA phages. A recent study also showed that for the *S. epidermidis* Type III-A CRISPR-Cas system, high levels of transcription across a DNA target made the system more reliant on the HD domain of Cas10 for anti-plasmid immunity^33^. Under those conditions, there may be increased R-loop formation that would allow Csm to access ssDNA more readily. Further studies will be needed to determine whether this is also relevant to anti-phage immunity, and what type of ssDNA substrate Csm would target in the cell. Csm may also be more closely related to Type V CRISPR-Cas enzymes than previously anticipated, as these also possess a target-activated ssDNA cleavage activity^38,39^. Lastly, our findings could guide strategies to repurpose Csm for detection or silencing of transcriptionally active loci in cells.

## Methods

### Plasmids and strains

To construct the vector, pCDF-5xT7-TtCsm, synthetic cassettes containing codon-optimized genes encoding for *T. thermophilus cas10/csm1* (GeneArt) and *csm2*-*csm5* (Genewiz) were cloned between the NcoI and AvrII sites of the pCDF-1b vector using Gibson assembly, overlap polymerase chain reaction (PCR), and restriction cloning^9^. Each gene was preceded by a T7 promoter, lac operator, and ribosomal binding site (RBS), and the *csm5* gene also included a sequence encoding for a 3C human rhinovirus (HRV) protease cleavage site and a C-terminal decahistidine tag (10xHistag). To construct pACYC-TtCas6-4xcrRNA4.5, the single CRISPR array in the pACYC-TtCas6-crRNA4.5 plasmid^9^ was replaced by a synthetic CRISPR array containing five repeats and four identical spacers corresponding to the 5^th^ spacer of the CRISPR-4 array from *T. thermophilus* HB8^7^(GeneArt). The pPS22 plasmid, which contains the *S. epidermidis* RP62A Type III-A CRISPR-Cas locus with an N-terminal hexahistidine tag on Csm2, was used for T7 promoter-driven expression of SepCsm^6^. All constructs were verified by Sanger sequencing. The ﻿*T. thermophilus* HB8rpoC::10H strain was used for expression and purification of His-tagged *T. thermophilus* RNAP^40^.

### Expression and purification of TthCsm

Expression and purification of TthCsm was performed as previously described with minor modifications^9^. Briefly, pCDF-5xT7-TtCsm and pACYC-TtCas6-4xcrRNA4.5 were transformed into *Escherichia coli* (*E. coli)* BL21(DE3) cells and grown in Terrific Broth (Novagen) to an optical density at 600 nm (OD600) of 0.6. Protein expression was induced by addition of IPTG (isopropyl-β-D-thiogalactoside) to 0.5 mM and expressed overnight at 16˚C^9^. The cells were lysed using an Avestin Emulsiflex homogenizer in buffer containing ﻿25 mM HEPES (pH 7.5), 150 mM KCl, 5% (v/v) glycerol, 10 mM imidazole, 1 mM TCEP (Tris(2-carboxylethyl)phosphine), and 0.01% (v/v) Triton X-100 supplemented with 1 mM PMSF (phenylmethylsulfonyl fluoride) and EDTA-free protease inhibitor cocktail tablets (Roche), and the lysate was clarified by centrifugation at 15,000 rpm in a JA-20 rotor for 30 min at 4˚C^9^. His-tagged TthCsm was isolated from the lysate using Ni^2+^-nitrilotriacetic acid (Ni^2+^-NTA) Superflow resin (Qiagen), and the resin was washed with Wash Buffer (50 mM HEPES (pH 7.5), 150 mM KCl, 5% (v/v) glycerol, 1 mM TCEP, 20 mM imidazole) containing 2 mM ATP and 10 mM MgCl_2_ to remove GroEL contaminants. This was followed by a second wash step using Wash Buffer without ATP or MgCl_2_. The protein was then eluted with buffer containing ﻿25 mM HEPES (pH 7.5), 150 mM KCl, 5% (v/v) glycerol, 1 mM TCEP and 300 mM imidazole^9^. The eluted fractions were pooled and the 10xHis tag on Csm5 was cleaved overnight at 4˚C with the 3C human rhinovirus (HRV) protease^9^. Imidazole was removed by dialysis against size-exclusion chromatography (SEC) buffer containing 25 mM HEPES (pH 7.5), 150 mM NaCl, 5% (v/v) glycerol, and 1 mM TCEP, and the sample was passed over Ni^2+^-NTA resin again to remove the cleaved tag and other impurities^9^. TthCsm was then purified by SEC on a Superose 6 10/300 GL column (GE Healthcare) in SEC buffer^9^. Fractions were screened by sodium dodecyl sulfate polyacrylamide gel electrophoresis (SDS-PAGE) and negative stain EM to obtain a homogeneous sample for EM and biochemical studies.

### Expression and purification of *T. thermophilus* RNAP

*T. thermophilus* RNAP containing a C-terminal 10xHis-tagged β´ subunit was purified from the *T. thermophilus* HB8rpoC::10H strain^40^. Cells were grown in Thermus Broth medium with 10 μg/mL kanamycin to an OD600 of 0.6-0.9, harvested by centrifugation, and disrupted by sonication in Buffer A2 (10 mM Tris-HCl (pH 8.0), 500 mM NaCl, 5% (v/v) glycerol, 1 mM TCEP) supplemented with 1 mM PMSF and 5 mM imidazole^40^. The lysate was clarified by centrifugation at 15,000 rpm in a JA-20 rotor for 40 min at 4 ˚C, and the His-tagged RNAP was purified using Ni^2+^-NTA Superflow resin (Qiagen)^40^. The column was sequentially washed with Buffer A2 containing 20, 40, and 80 mM imidazole, and bound protein was eluted with Buffer A2 containing 200 mM imidazole^40^. The eluted protein was further purified by SEC on a Superdex 200 Increase 10/300 GL column (GE Healthcare) in buffer containing 20 mM Tris (pH 8.0), 200 mM KCl, 0.5 mM EDTA, 5% (v/v) glycerol, and 1 mM TCEP, flash frozen in aliquots in liquid nitrogen, and stored at −80 °C.

### Preparation of DNA and RNA oligonucleotides for TEC assembly and pull-downs

RNA oligonucleotides > 60 nts in length were prepared by in vitro transcription of a DNA template using T7 RNA polymerase. Templates were prepared by annealing a short T7 oligonucleotide (Supplementary Table 1, T7 oligo) to the promoter region of a template ssDNA containing a sequence complementary to the desired RNA sequence. RNA oligonucleotides < 60 nts in length were ordered from Integrated DNA Technologies (IDT). DNA substrates were ordered from IDT. All oligonucleotides were purified by denaturing urea PAGE in 0.5× TBE (Tris-borate-EDTA) buffer and ethanol-precipitated prior to use, except for the desthiobiotinylated DNA, which was HPLC-purified by IDT. All oligonucleotide sequences are given in Supplementary Table 1.

### Pull-down assays with TthCsm and TEC

To form the nucleic acid scaffold, DNA oligonucleotides corresponding to the nontemplate strand of DNA (Supplementary Table 1, TthNTS1, TthNTS2) and desthiobiotinylated template DNA of the TEC (Supplementary Table 1, TthTS1, TthTS2) and the in vitro transcribed ssRNA oligonucleotide were added at a 1:1:1 ratio at a final concentration of 10-20 µM in HN100 buffer (40 mM HEPES (pH 7.9), 100 mM NaCl, 1 mM TCEP, and 1 mM EDTA) and heated at 95 ˚C followed by slow cooling on a benchtop for 15 min and incubation on ice for 5 min. The *T. thermophilus* RNAP (0.67 µM) was added to 2 µM nucleic acid scaffold in HN100 with 10 mM MgCl_2_, incubated at 37 ˚C for 15 min, 10 min at room temperature, and then kept on ice until use. Assembled TECs with a desthiobiotinylated template strand were immobilized on Streptavidin Mag Sepharose (GE Healthcare) for 1 h, and washed with HN100 buffer. TthCsm (3 µM) was added and incubated for 25 min at 65 ˚C, and unbound complexes were washed away with HN100 buffer. Bound complexes were eluted with 40 mM HEPES (pH 7.9), 76 mM NaCl, 1 mM EDTA, 1 mM TCEP, 2% (v/v) glycerol, and 5 mM biotin at 37 ˚C for 30 min. Elutions were analyzed by SDS-PAGE, and a PageRuler molecular weight marker (Thermo Scientific) was included for comparison. For experiments in which target RNA was added in *trans*, a 50-nt ssRNA oligonucleotide containing a 40-nt sequence complementary to the crRNA guide region^7,9^ was added at the same time as TthCsm at an equimolar concentration (3 µM).

### Electrophoretic mobility shift assays (EMSAs)

Varying concentrations of TthCsm were incubated with 0.5 nM ^32^P-radiolabeled ssDNA **(**Supplementary Table 1, ssRNA, NC) at 65˚C for 20 min^9^. The binding buffer contained 25 mM Tris (pH 7.9), 40 mM KCl, 5% (v/v) glycerol, 1 mM TCEP, and 1 mM EDTA. Binding reactions were analyzed by 6% native PAGE at 4 ˚C in 0.5× TBE buffer, and the ^32^P-radiolabeled oligonucleotide was visualized by phosphorimaging^9^.

### Negative stain EM data collection and analysis

Eluted TthCsm-TEC complexes from the pull-downs were diluted 1:20 to a final concentration of ~30 nM and negatively stained with a 2% (w/v) solution of uranyl acetate (Electron Microscopy Sciences) on glow-discharged holey carbon-coated EM copper grids covered with a thin layer of continuous carbon^41^. The negatively stained specimen was then mounted onto a transmission electron microscope holder and examined using an FEI Tecnai T12 electron microscope operated at 120 kV. Digital micrographs of the specimen were automatically collected using Leginon at a nominal magnification of 49,000× with a pixel size of 2.18 Å at the specimen level. The defocus was in the range of –0.8 to –1.5 μm, and the total accumulated dose at the specimen was ~58 electrons/Å^2^. To enrich the Euler angle distribution, half of the data set was collected with a tilt angle of 30˚. Particles were picked using DoGpicker, low-pass filtered to 10 Å, normalized, binned by 2, and then subjected to reference-free 2D classification in Appion^42^. Then, 17 representative 2D class averages containing both TthCsm and RNAP were imported into EMAN2 to generate an initial 3D model using the common line method^43^. Good particles sorted by 2D classification were further subjected to 3D classification and refined against the initial model in Relion 1.4^44^. 3D classes containing both the TthCsm and well-defined RNAP densities were subsequently used for localized refinement based on the TthCsm density, and the localized refinement metadata were then used for alignment-free 3D classification, which generated well-defined EM maps for fitting of TthCsm and RNAP structures. Different 3D classes were aligned based on the TthCsm density in UCSF Chimera for presentation^45^.

### Cryo-EM sample preparation and data collection

For cryo-EM sample preparation of TthCsm bound to RNA, TthCsm (3 µM) was mixed with a 50-nt complementary RNA target (Supplementary Table 1, RNA 4.5) and a ssDNA substrate (Supplementary Table 1, ssDNA, NC)^9^ in buffer containing 25 mM HEPES (pH 7.9), 40 mM KCl, 1 mM TCEP, 1 mM EDTA, and 5% (v/v) glycerol, and incubated at 65 ˚C for 10 min. Unbound nucleic acids were separated from the substrate-bound TthCsm complex on a Superose 6 10/300 GL column. Peak fractions were analyzed on a denaturing urea polyacrylamide gel in 0.5× TBE buffer with SYBR Gold staining (Thermo Fisher Scientific). The TthCsm peak was collected, concentrated to ~1.5 µM, and supplemented with additional ssDNA at a final concentration of 2 µM. The sample quality was initially evaluated by negative stain EM. Then, the sample was then diluted ~1:3 with buffer containing 25 mM HEPES (pH 7.9), 40 mM KCl, 1 mM EDTA, and 1 mM TCEP, and 3.6 μl droplets of the sample were placed onto glow-discharged C-flat grids with 2 μm holes and 2 μm spacing between holes covered by thin carbon film (Protochips Inc.). The grids were rapidly plunged into liquid ethane and data were acquired using an FEI Titan Krios electron microscope operated at 300 keV, at a nominal magnification of 24,500 × (1.08 Å pixel size), with a defocus range of *–*1.0 to –3.0 μm. A total of 4,856 micrographs were recorded using SerialEM on a Gatan K2 Summit direct electron detector operated in super-resolution mode^46^. For each micrograph, we collected 7.5 s exposures as 30 dose-fractionated frames of 250-ms, at a dose rate of 6 e^-^/Å^2^ per second.

For the TthCsm-TEC complex, the eluted complex from the pull-down was used directly for cryo-EM sample preparation. For grid preparation, 3.6 μl droplets of the sample were placed onto glow-discharged C-flat grids with 2 μm holes and 2 μm spacing between holes, covered by thin carbon film (Protochips Inc.). The grids were blotted for 4.5 seconds with a blot force of 12, and rapidly plunged into liquid ethane using a FEI Vitrobot Mark IV maintained at 8 ˚C and 100% humidity. Data were acquired using FEI Titan Krios electron microscope operated at 300 keV, at a nominal magnification of 18,000 × (1.22 Å pixel size), with defocus range of -1.0 to -3.0 μm. A total of 6,502 micrographs were recorded using SerialEM on a Gatan K2 Summit direct electron detector operated in super-resolution mode. For each micrograph, we collected a 6.5 s exposure as 32 dose-fractionated frames of 200 ms each at a dose rate of 6.7 e^-^/Å^2^ per second.

### Cryo-EM data processing and reconstruction

For the RNA-bound TthCsm data set, the 30 frames (we skipped the first and last frames) of each image stack were aligned, decimated, and summed and dose-weighted using Motioncor2^47^. CTF values of the summed micrographs were determined using CTFFIND4 and then applied to the dose-weighted, summed micrographs for further processing^48^. Particle picking for the complete dataset was carried out using Gautomatch (http://www.mrc-lmb.cam.ac.uk/kzhang/) with templates from negative staining 2D class-averages (TthCsm only). A total of 670,651 particles were selected and imported into CryoSparc for 2D analysis^49^. All the particles that belonged to a bad class, as well as particles in classes corresponding to the preferred orientation (60%) were discarded. The rest, 226,621 particles, were used for 3D *ab initio* modeling. Particles belonging to good classes were further classified into 3 classes and those in the best class were further refined to 3.8 Å. Local resolution was calculated using Relion 2.0^50^. The reported resolution was based on the gold standard FSC criterion using two independent half-maps.

For the TthCsm-TEC data set, the 32 frames (we skipped the first and last 2 frames) of each image stack were aligned, decimated, and summed and dose-weighted using Motioncor2^47^. CTF values of the summed micrographs were determined and applied to micrographs as described above^48^. Particle picking for the complete dataset was carried out using Gautomatch with templates from the 2D negative stain class averages (TthCsm-TEC). A total of 381,092 particles were selected and imported into CryoSparc for 2D classification.

### Model building and validation

The homologous models of *T. onnurineus* Csm1 (PDB ID: 4UW2 [10.2210/pdb4UW2/pdb]), *Thermotoga maritima* Csm2 (PDB ID: 5AN6 [10.2210/pdb5AN6/pdb]), and the *Methanococcus jannaschii* Csm3-Csm4 complex (PDB ID: 4QTS [10.2210/pdb4QTS/pdb]) were used as templates for TthCsm subunit model building in Swiss-model^51–54^. Csm2 and Csm3 were independently built, fit into the EM map, and then used to generate multiple copies of these subunits in the complex. The crRNA and target RNA models were manually built in *Coot* based on the EM density and using segments of the crRNA:ssDNA hybrid in a Type III-B complex (PDB ID: 3X1L [10.2210/pdb3X1L/pdb]) as a template^26,55^. An intact RNA-bound Csm initial model was then generated by assembling Cas10/Csm1, four copies of Csm2, six copies of Csm3, Csm4, crRNA and target RNA models. The initial model was manually edited against the EM map in *Coot*, including adjustment of backbone, deletion of invisible loops and building of unassigned protein densities. The manually rebuilt model was subjected to PHENIX real space refinement (global minimization, simulated annealing, morphing, local grid search and ADP refinement) with Ramachandran, rotamer, secondary structure, and nucleic-acid restraints against the EM map^56^. The final model was validated using *Molprobity*^57^. Structural analysis was performed in *Coot* and figures were prepared using UCSF Chimera. Data collection and refinement statistics are in Supplementary Table 2.

Modeling of the *Thermus* TEC was done in UCSF Chimera using structures of the *T. thermophilus* transcription elongation complex (PDB ID: 2O5I [10.2210/pdb2O5I/pdb]), which contains a downstream DNA duplex and an RNA transcript spanning the length of the exit channel, and the closely related *Thermus aquaticus* transcription initiation complex, which contains a complete, 13-nt DNA bubble (PDB ID: 4XLN [10.2210/pdb4XLN/pdb])^16,19^.

### Cleavage assays with TthCsm and *T. thermophilus* TECs

The TthCsm-TEC pull-down elution (2 µl) was diluted to 10 µl with HN100 buffer. Reactions were initiated by adding 5 mM MgCl_2_ to allow RNA cleavage or 5 mM MnCl_2_ to allow both ssDNA and RNA cleavage. Following incubation at 65 ˚C for 30 min, reactions were quenched with 1 volume of 2X Gel Loading Buffer II (Invitrogen). Reactions were analyzed by denaturing 15% urea PAGE in 0.5× TBE buffer and SYBR gold stain (Thermo Fisher) was used to visualize ssDNA and ssRNA.

For radioactive cleavage assays with the TEC, assembly of the TEC and pull-downs with TthCsm were performed as described above, except that a trace amount of nontemplate DNA was 5´-end-labeled with [γ-^32^P]-ATP (Perkin-Elmer) using T4 polynucleotide kinase (PNK; New England Biolabs) and incorporated into the TEC. For reactions with free nontemplate strand substrate, 0.8 µM of nontemplate DNA (Supplementary Table 1, TthNTS1 or TthNTS2) was mixed with 0.2 µM TthCsm and 0.2 µM of the target RNA transcript. The same was done for reactions using the empty R-loop, except 0.8 µM of nontemplate DNA was annealed with an equimolar amount of the template strand (Supplementary Table 1, TthTS1 or TthTS2), forming a DNA mismatch bubble. Cleavage reactions (20 µl) were initiated by addition of 5 mM MnCl_2_. A 5´-end-labeled ssDNA ladder was generated using truncations of the nontemplate strand DNA.

### Expression and purification of SepCsm

SepCsm was expressed and purified essentially as described^6^. Briefly, the pPS22 plasmid was transformed into Rosetta 2 BL21(DE3) cells, and 10 L of cells were grown in Terrific Broth to an OD600 of 0.6^6^. Protein expression was induced by addition of IPTG to 0.3 mM, followed by growth at 17 ˚C for 16 h^6^. The cells were then harvested by centrifugation, flash frozen with liquid nitrogen, and stored at -80 ˚C^6^. For purification, cell pellets were thawed and resuspended in Buffer A (50 mM Tris-HCl (pH 7.5), 350 mM NaCl, 200 mM Li_2_SO_4_, 20% (w/v) sucrose, 10 mM imidazole) supplemented with EDTA-free protease inhibitors (Roche), 0.1% (v/v) Triton X-100, and 0.1 mg/ml lysozyme^6^. After incubation on ice for 1 h, the cells were sonicated and the cell lysate was clarified by centrifugation at 15,000 rpm in a JA-20 rotor for 40 min at 4˚C^6^. The clarified lysate was then incubated with 5 ml of Ni^2+^-nitrilotriacetic acid (Ni^2+^-NTA) affinity resin (Qiagen) to capture His-tagged SepCsm^6^. The resin was washed with Buffer A, and then sequentially with IMAC buffers (50 mM Tris-HCl (pH 7.5), 250 mM NaCl, 10% (v/v) glycerol) containing 15 mM imidazole and 50 mM imidazole. The protein was then eluted by stepwise addition of two 5-ml volumes each of 100, 200, 350 and 500 mM imidazole in IMAC buffer^6^. Fractions containing SepCsm were diluted and supplemented with EDTA to prevent aggregation during dialysis, and then dialyzed against 4 L of dialysis buffer (50 mM Tris (pH 7.5), 50 mM NaCl, and 10% (v/v) glycerol)^6^. The complex was then subjected to anion exchange chromatography on a 1 ml Resource Q column (GE Healthcare) using a linear gradient of 0.05–2 M NaCl over 20 column volumes. Peak fractions containing the complex were then subjected to SEC on a Superdex 200 10/300 GL column (GE Healthcare) in Buffer B (﻿50 mM Tris-HCl (pH 7.5), 150 mM NaCl, 5% (v/v) glycerol)^6^. Fractions containing purified SepCsm were pooled and flash frozen in aliquots using liquid nitrogen and stored at -80 ˚C^6^. Protein concentration was determined with the Quickstart Bradford Protein Assay (Biorad), using bovine serum albumin (BSA) standards (Biorad). SDS-PAGE was used to assess protein purity and the Precision Plus protein ladder (Biorad) was used for size comparison. Verification of crRNA content was performed by phenol-chloroform extraction of the crRNAs from the complex, followed by 5´-^32^P-radiolabeling of the crRNAs with T4 PNK (New England Biolabs), denaturing 14% urea PAGE analysis, and phosphorimaging^6^.

### Co-transcriptional DNA and RNA cleavage assays with SepCsm

These assays were performed as described, unless otherwise indicated^6^. Briefly, the 90-nt long nontemplate DNA (Supplementary Table 1, PS365) or the 10-nt long RNA primer (Supplementary Table 1, EC primer 1) was 5´-^32^P-radiolabeled with T4 PNK (New England Biolabs), PAGE-purified, ethanol precipitated in 1 M ammonium acetate, and resuspended in 1× TE buffer (10 mM Tris (pH 8.0), 1 mM EDTA)^6^. TECs were assembled in a stepwise manner in 1× transcription buffer (20 mM Tris-HCl (pH 7.9), 40 mM KCl, 5 mM MgCl_2_, and 2.5 mM DTT (dithiothreitol))^6^. Excess RNA primer (200 nM) was annealed to template DNA (100 nM; Supplementary Table 1, PS364) by heating to 65 ˚C and cooling slowly to 25 ˚C, followed by binding of 1.5 µl *E. coli* core RNAP enzyme (New England Biolabs) at 25 ˚C, and incubation of the nontemplate DNA strand (250 nM) at 37 ˚C^6^. In experiments with a 0.8:1 ratio of NTS:TS, a smaller amount of the nontemplate DNA (80 nM) was used. 1× transcription buffer was added to bring the TECs to a final concentration of 100 nM. For co-transcriptional DNA and RNA cleavage assays, TECs (10 nM) were incubated for 10 min at 37 ˚C with 2.5 mM ribonucleotides prior to addition of SepCsm to a final concentration of 15 ng/µl^6^. Where indicated, SepCsm was incubated with the TECs at 37˚C for 10 min before ribonucleotides were added. Samples were taken out and quenched at indicated timepoints with proteinase K (New England Biolabs) and 20 mM EDTA, phenol-chloroform extracted, ethanol precipitated, and resuspended in 90% (v/v) formamide, 50 mM EDTA, with trace amounts of bromophenol blue and xylene cyanol as gel tracking dyes^6^. DNA cleavage samples were analyzed on a 12% denaturing urea polyacrylamide gel and RNA cleavage samples were analyzed on a 14% denaturing urea polyacrylamide gel^6^.

An RNA Decade™ ladder (Ambion) was used for size comparison of RNA cleavage reactions^6^. A 5´-radiolabeled ssDNA marker was made by truncating the nontemplate ssDNA (Supplementary Table 1, PS365) from its 3´end to the indicated lengths (20-90 nts). Sequences of all oligonucleotides used are given in Supplementary Table 1.

### RNA transcription with TECs

TECs were assembled with *E. coli* RNAP as described above and diluted to a concentration of 10 nM in 1× transcription buffer. After pre-warming the TECs at 37˚C for 10 min, 2.5 mM ribonucleotides were added to initiate transcription. Samples were collected at 2, 5, 10, 40, and 70 min, and quenched with proteinase K and 20 mM EDTA. Removal of proteins by phenol-chloroform and ethanol precipitation prior to analysis on a denaturing 14% urea polyacrylamide gel was performed in the same way as for co-transcriptional DNA and RNA cleavage samples.

### Reporting Summary

Further information on research design is available in the Nature Research Reporting Summary linked to this article.

## Supplementary information

Supplementary Information

Reporting Summary

Source data

## Data Availability

The cryo-EM map and model of RNA-bound TthCsm have been deposited in the Electron Microscopy Data Bank (EMDB) under accession codes EMD-0454 and PDB-6O1O, and the negative stain EM map of TthCsm-TEC has been deposited in the EMDB under accession code EMD-0455. The pPS22 plasmid is available from Addgene (#70038). The pCDF-5xT7-TtCsm (Addgene #128572) and pACYC-TtCas6-4xcrRNA4.5 (Addgene #127764) plasmids will be deposited in and available from Addgene. All other relevant materials will be available from the authors upon request. Uncropped gel images underlying Figs. 1c-d, 4a-b, 5b-d and Supplementary Figs. 1, 3a-b, 6, 7a-d, 8 are available online in the Source Data file.
