# Supplementary material for: Target preference of Type III-A CRISPR-Cas complexes at the transcription bubble
Source: Nat Commun. 2019 Jul 5;10:3001. doi: 10.1038/s41467-019-10780-2 (PMC6611850; doi:10.1038/s41467-019-10780-2)
Supplement: Supplementary file 1 — Supplementary Information [file 41467_2019_10780_MOESM1_ESM.pdf]

Supplementary Information

for

Target preference of Type III-A CRISPR-Cas complexes at the transcription bubble

T.Y. Liu, J.-J. Liu *et al.*

This file contains:

Supplementary Figure 1

Supplementary Figure 2

Supplementary Figure 3

Supplementary Figure 4

Supplementary Figure 5

Supplementary Figure 6

Supplementary Figure 7

Supplementary Figure 8

Supplementary Table 1

Supplementary Table 2

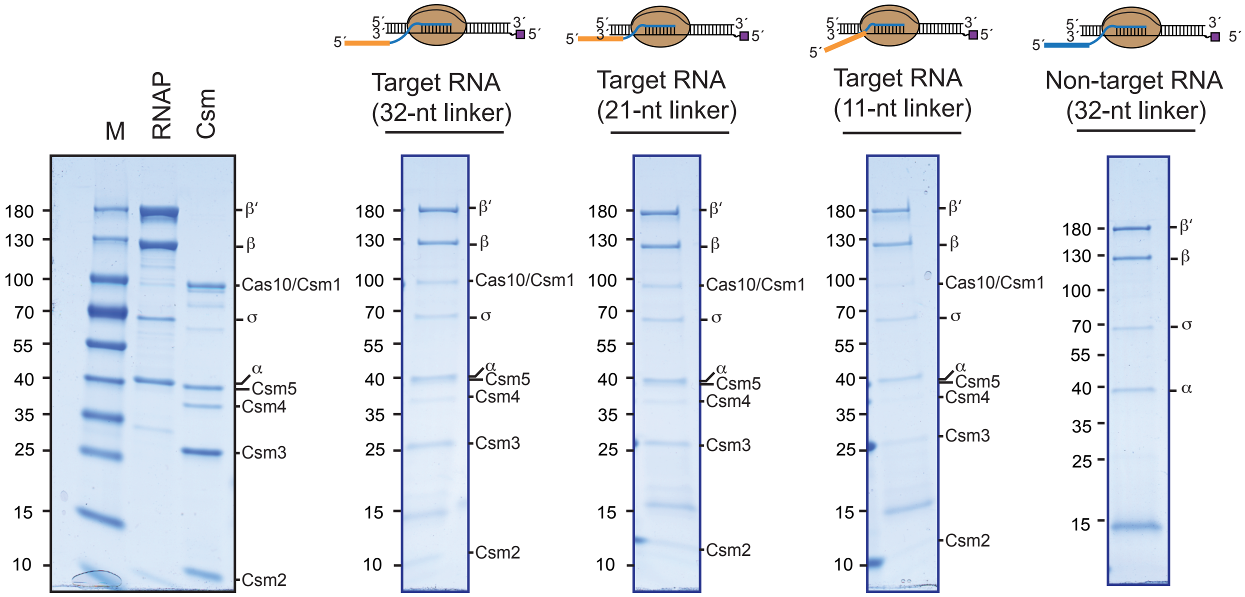

Supplementary Figure 1. Pulldown of TthCsm with TECs containing nascent RNA of different lengths, related to Fig. 1.
Pulldowns were performed as in **Fig. 1**, but with TECs containing RNAs with increasingly shorter sequences between the 5′ crRNA-complementary sequence and the 3′ template DNA-complementary sequence (21-nt and 11-nt linkers). The gel from **Fig. 1c** showing purified *T. thermophilus* RNAP and TthCsm is included on the left for comparison. The sizes corresponding to the molecular weight marker (M) are indicated in kilodaltons. The pulldown with TECs containing a target RNA in **Fig. 1d** is shown here as Target RNA (32-nt linker) for comparison. The pulldown from **Fig. 1d** using TECs containing an RNA lacking the crRNA-complementary sequence is shown here in the rightmost panel as Non-target RNA (32-nt linker). All uncropped gel images are provided in the Source Data file.

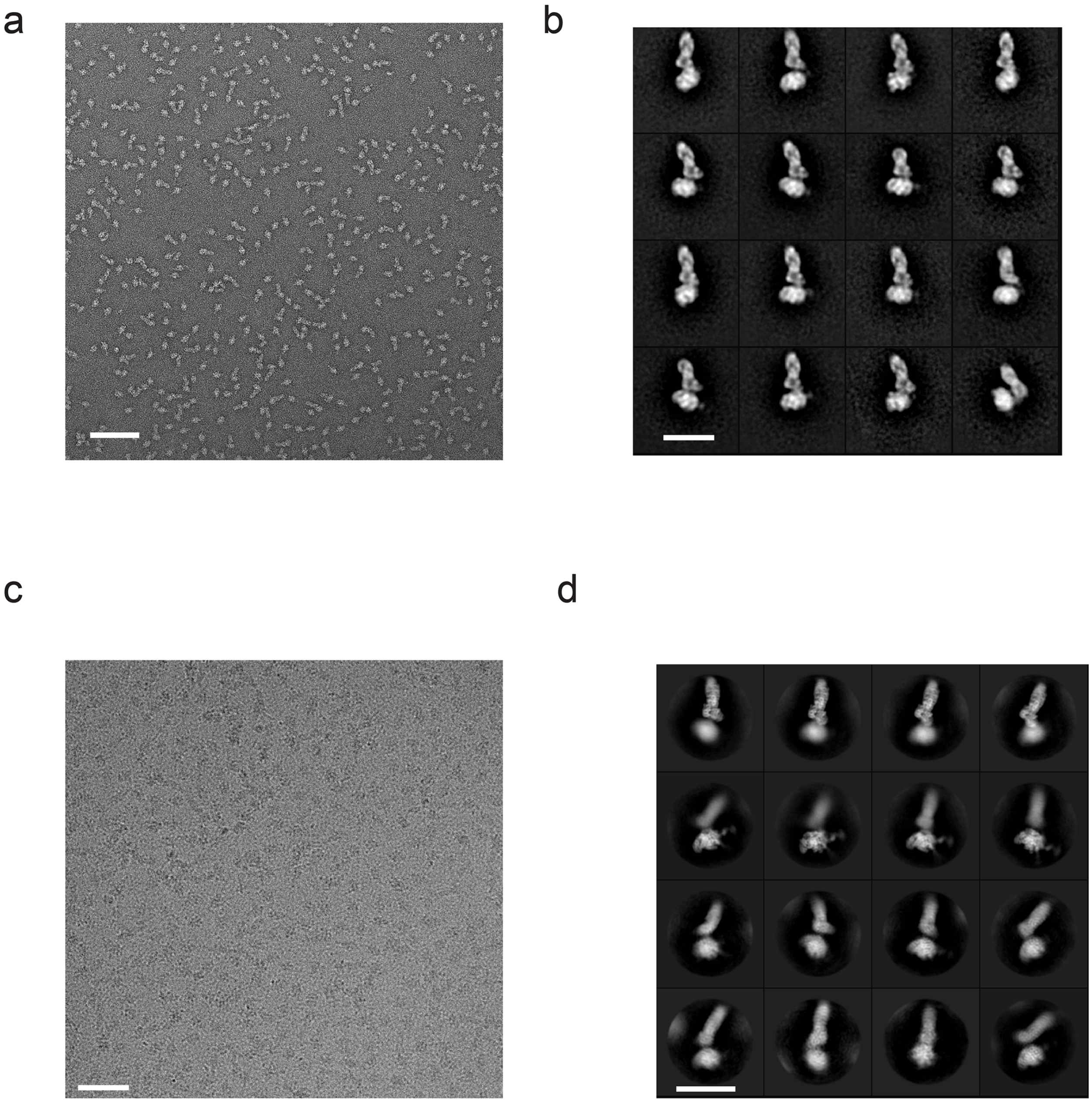

Supplementary Figure 2. Negative-stain EM and cryo-EM analysis of TthCsm-TEC complex, related to Fig. 2.

a Representative negative-stain EM micrograph of TthCsm-TEC. The scale bar is 100 nm. **b** 2D class averages of the negatively stained TthCsm-TEC complex. 16 representative class averages are shown with a scale bar of 30 nm. **c** Representative cryo-EM micrograph of TthCsm-TEC. The scale bar is 50 nm. **d** As in **b**, but for the cryo-EM sample of TthCsm-TEC. The scale bar is 30 nm.

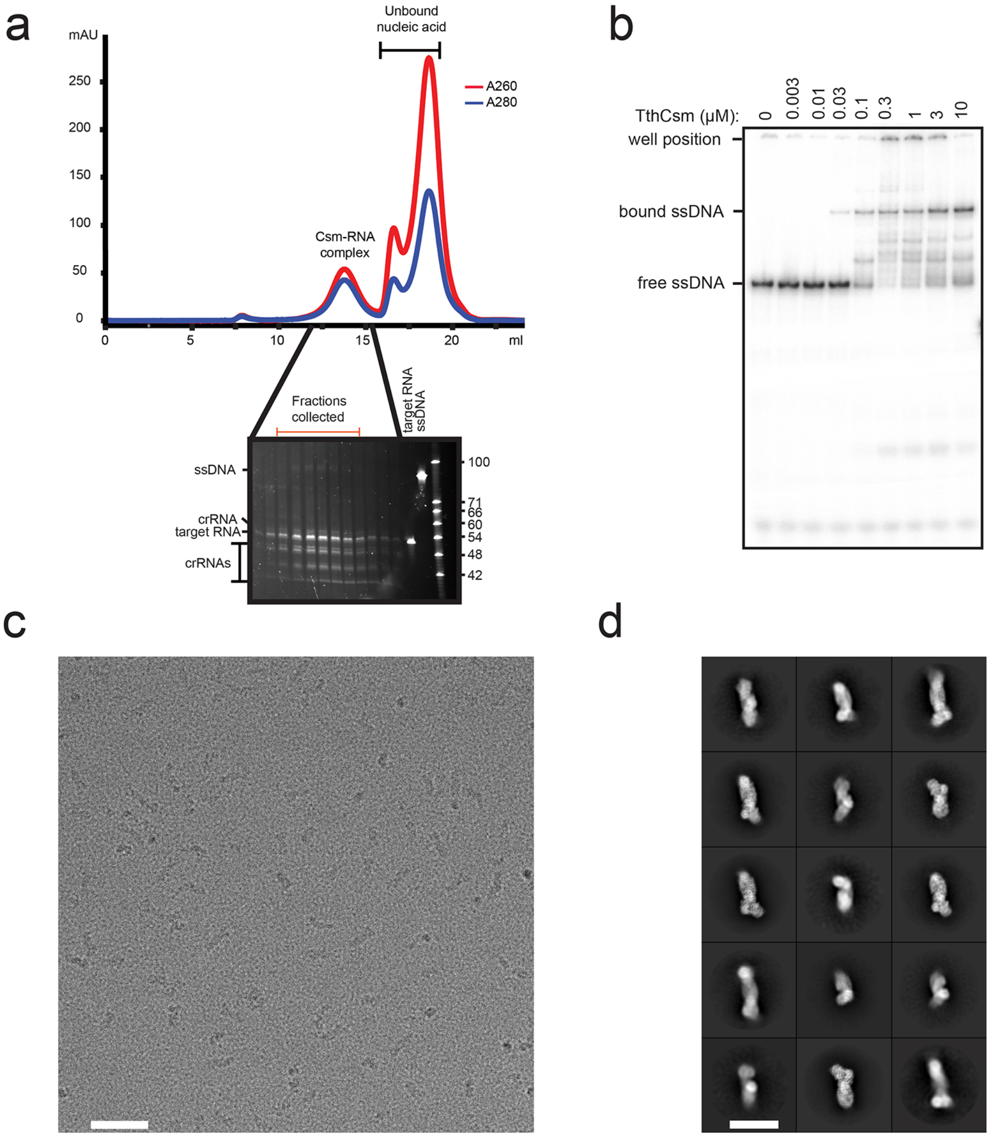

Supplementary Figure 3. Preparation and cryo-EM analysis of target RNA-bound TthCsm, related to Fig. 3.

a Size-exclusion chromatography of the TthCsm incubated with a complementary RNA target and a noncomplementary ssDNA oligonucleotide on a Superose 6 column. Analysis of the peak fractions by denaturing PAGE and SYBR gold staining are shown in the panel below. The nucleic acids corresponding to TthCsm crRNAs, target RNA, and ssDNA are indicated. An ssDNA ladder was loaded in the rightmost lane for size comparison. **b** Electrophoretic mobility shift assay of TthCsm with 5′-^{32}P-labeled noncomplementary ssDNA used in **a.** **c** Representative cryo-EM micrograph of the target ssRNA-bound TthCsm in **a** collected on a Titan Krios equipped with a K2 camera. The scale bar is 50 nm. **d** 2D class averages of the target RNA-bound TthCsm obtained by single particle analysis. 15 representative classes are shown with a scale bar of 20 nm. Uncropped gel images for **a** and **b** are provided in the Source Data file.

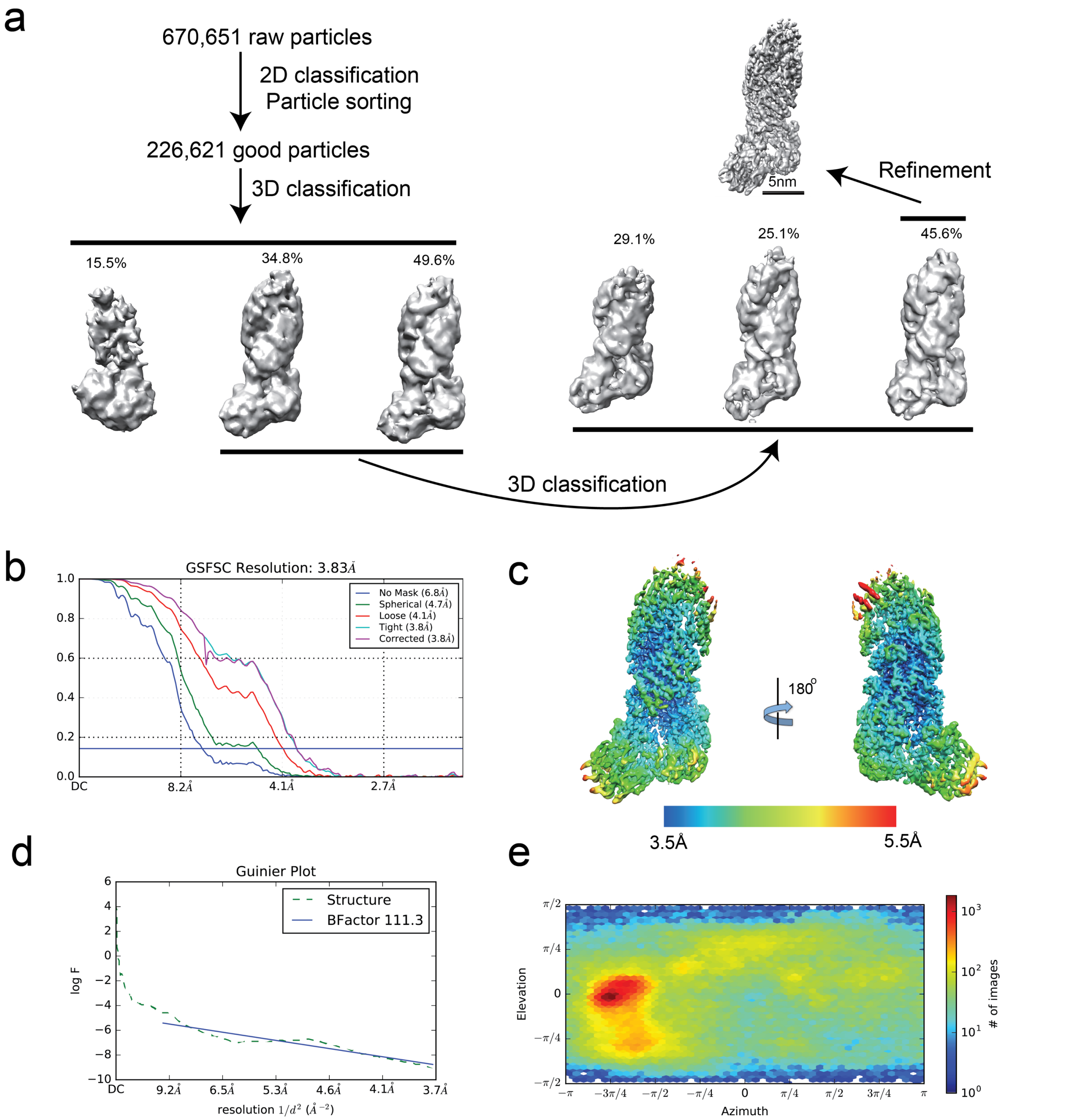

Supplementary Figure 4. 3D cryo-EM reconstruction and data processing workflow of TthCsm bound to target RNA, related to Fig. 3.

a EM analysis pipeline for target RNA-bound TthCsm. A total of 626,621 particles were selected and imported into CryoSparc for 2D analysis. After discarding particles present in bad classes or in classes corresponding to the preferred orientation, 226,621 particles were used for 3D *ab initio* modeling into three classes. Particles belonging to the second and third classes were combined and further classified in to three classes. The particles belonging to the third class were further refined. **b** Fourier shell correlation (FSC) curve calculated using two independent half maps. **c** Local resolution map of the refined model in two orientations. The resolution range is from 3.5 Å to 5.5 Å. **d** Guinier Plot for the refined model. The B-factor used for map sharpening is shown. **e** Euler angle distribution of the refined particles. Panels **b, d** and **e** are directly taken from the standard output of CryoSparc.

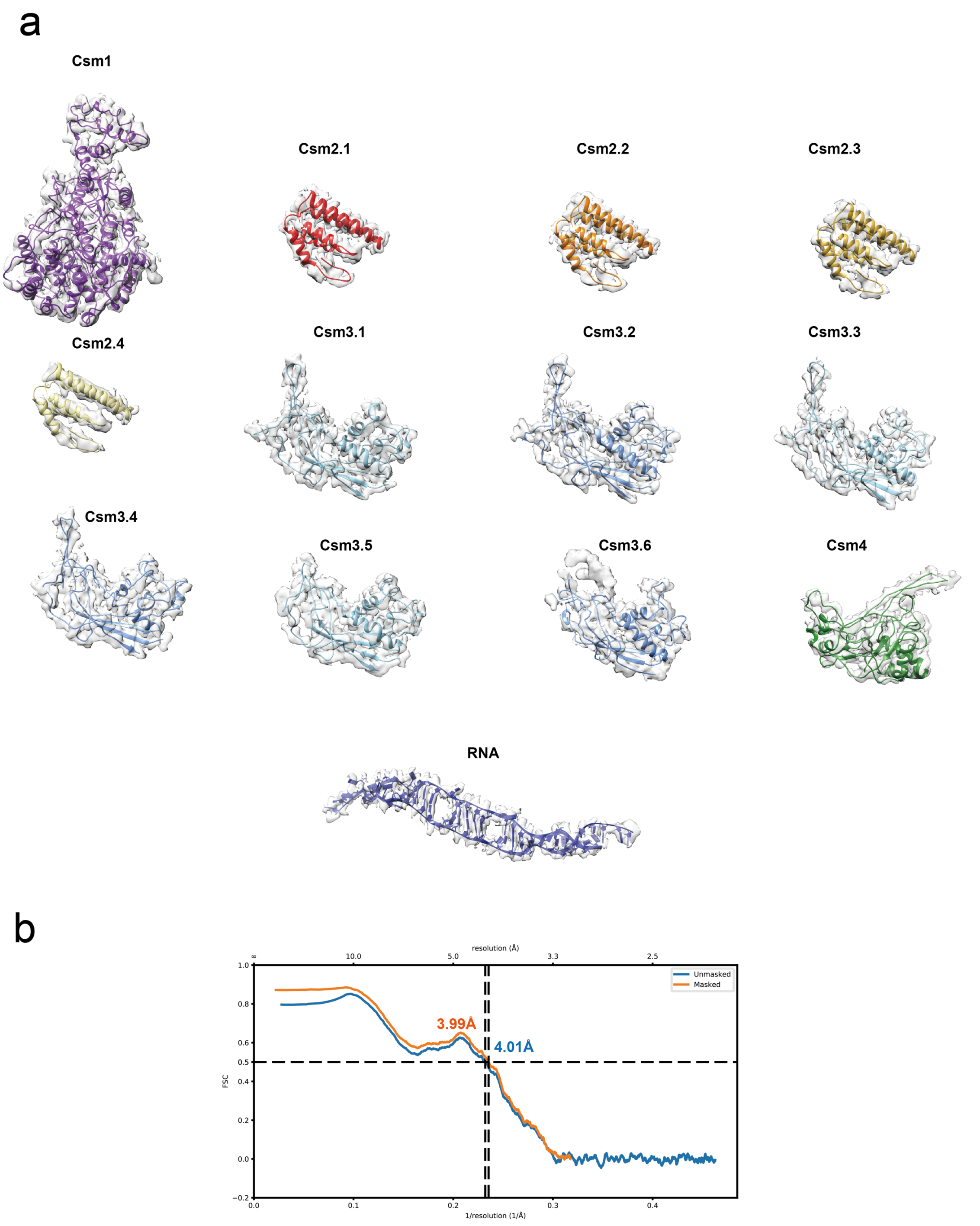

Supplementary Figure 5. Details and validation of the atomic model of TthCsm bound to a complementary target RNA, related to Fig. 3.
a The atomic models of the crRNA:target RNA hybrid, and the individual TthCsm subunits are shown, fitted into the corresponding regions of the EM density map. The atomic models are shown in a cartoon representation with same color scheme as in **Fig. 3a,** except that both strands of the crRNA:target RNA hybrid are colored dark blue. The EM map is shown as a solid density with 70% transparency. **b** FSC of the EM density map against the atomic model for TthCsm-target RNA complex.

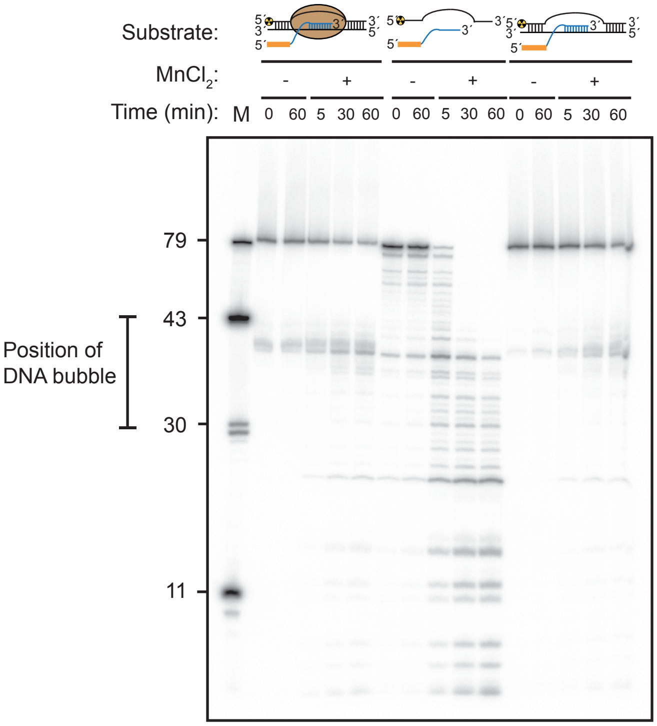

Supplementary Figure 6. Testing TthCsm's ssDNA cleavage activity at the TEC, related to Fig. 4. As in **Fig. 4b**, but with a TEC containing a 30-nt, rather than 11-nt, duplex 5´ to the unpaired DNA bubble. A ssDNA ladder was loaded in the leftmost lane (M), with the lengths indicated in nucleotides. The position of the unpaired DNA bubble is indicated. The uncropped gel image is provided in the Source Data file.

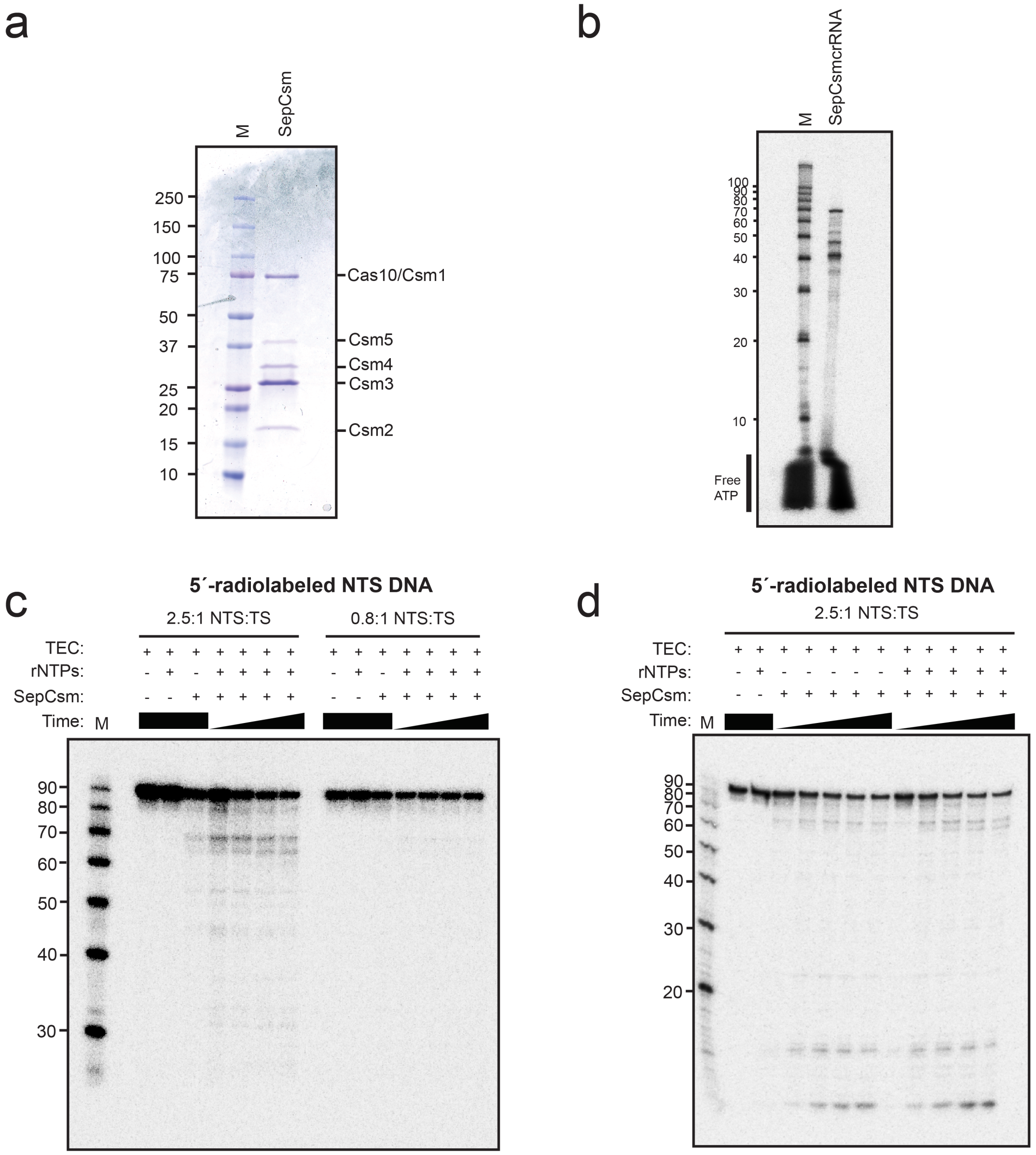

Supplementary Figure 7. Characterization of purified SepCsm, and co-transcriptional DNA cleavage by SepCsm, related to Fig. 5.

a Purified SepCsm was analyzed by SDS-PAGE to verify the presence of all five subunits. A molecular weight marker is shown in the leftmost lane. **b** The crRNAs of SepCsm were phenol-chloroform extracted, 5´-radiolabeled with T4 PNK, and analyzed by denaturing 14% PAGE. An RNA Decade™ marker (M) was loaded in the leftmost lane. **c** Co-transcriptional DNA cleavage by SepCsm was performed as in **Fig. 5c**, but after running the gel for a longer period of time to allow smaller fragments to run off the bottom of the gel, and obtain better resolution of the cleavage products ~70 nt. The DNA substrates and NTS:TS ratios used are the same as in **Fig. 5c**. A ssDNA ladder is loaded in the leftmost lane (M). **d** Co-transcriptional DNA cleavage by SepCsm. As in **Fig. 5c**, but with samples taken at 5, 30, 60, 90, 120 min for all reactions with SepCsm added. Uncropped gel images for **a-d** are provided in the Source Data file.

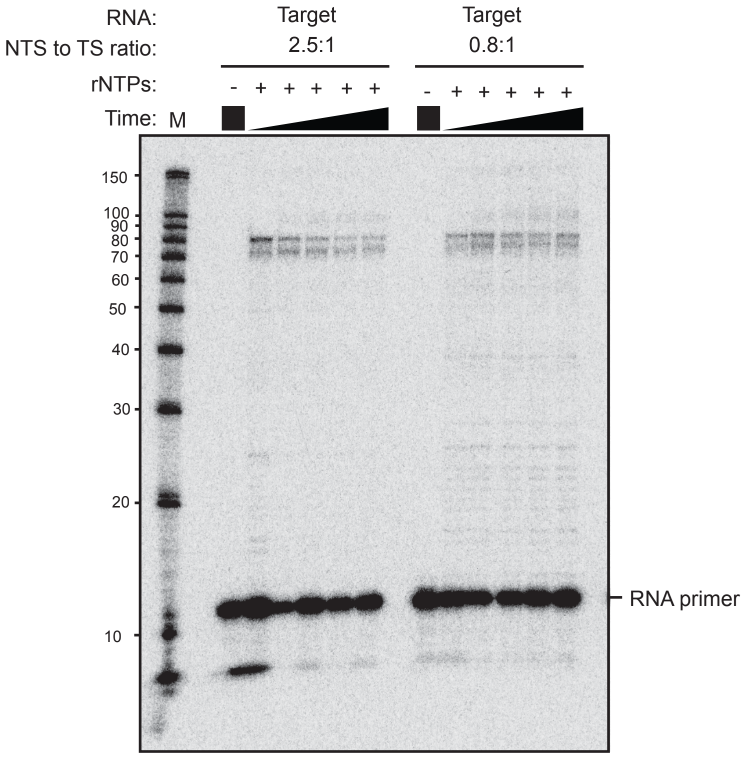

Supplementary Figure 8. Transcriptional activity of TECs, related to Fig. 5.
The transcriptional activity of TECs used in **Fig. 5c** was determined with a radioactive ^{32}P label on the 5´-end of the RNA primer instead of the nontemplate DNA strand. No SepCsm was added in these reactions. TECs with either an NTS:TS ratio of 2.5:1 or 0.8:1 were incubated at 37˚C with rNTPs (ribonucleotides) and samples were taken at 2, 5, 10, 40, 70 min and analyzed by denaturing 14% urea PAGE. A sample of a reaction lacking rNTPs was also taken at 70 min. An RNA Decade™ marker (M) was loaded in the leftmost lane. The uncropped gel image is provided in the Source Data file.

Supplementary Table 1. DNA and RNA oligonucleotide sequences

**TthCsm-TEC assembly and cleavage substrates and markers**		
TthNTS1	DNA	5'-CTCCTCCCTCTTCACTTCCTGTCTCTTCTCTCCTGGTCGTGTTGTCTGGAATTCCTCTCC
TthTS1	DNA	5'-desthiobiotinTEG/GGAGAGGAATTCCAGACAACACGACCAGGAGAGAAGGATGTCCGGCAAGAGAGGGAGGAG
TthNTS2	DNA	5'-GCCTCCTTGTCCTCGCTCTCTCCTCCCTCTTCACTTCCTGTCTCTTCTCTCCTGGTCGTGTTGTCTGGAATTCCTCTCC
TthTS2	DNA	5'-desthiobiotinTEG/GGAGAGGAATTCCAGACAACACGACCAGGAGAGAAGGATGTCCGGCAAGAGAGGGAGGAGAGAGCGAGGACAAGGAGGC
TthNTS1_11	DNA	5'-CTCCTCCCTCT
TthNTS1_17	DNA	5'-CTCCTCCCTCTCTTGCC
TthNTS1_24	DNA	5'-CTCCTCCCTCTCTTGCCGGACATC
TthNTS2_30	DNA	5'-GCCTCCTTGTCCTCGCTCTCTCCTCCCTCT
TthNTS2_43	DNA	5'-GCCTCCTTGTCCTCGCTCTCTCCTCCCTCTCTTGCCGGACATC
**T7 *in vitro* transcription templates**		
T7oligo	DNA	5'-TAATACGACTCACTATA
RNA3NT	DNA	5'-TGTCCGGCATGTTGGTGTTCTTCTCGTTTCGTGCTGTGCTTCCAGACCGTTCAGCTGGATATTACGGCCTTTTTAAAGACCCCTATAGTGAGTCGTATTA
RNA3T	DNA	5'-TGTCCGGCATGTTGGTGTTCTTCTCGTTTCGTGCTGTGCTTCCGTAGATAAGGCGCCCGGGGACGACCACGTCAAGGCGCACCTATAGTGAGTCGTATTA
RNA2T	DNA	5'-TGTCCGGCATGTTGGTGTTGTGCTGTGCTTCCGTAGATAAGGCGCCCGGGGACGACCACGTCAAGGCGCACCTATAGTGAGTCGTATTA
RNA1T	DNA	5'-TGTCCGGCATTTACGTTTTTCCGTAGATAAGGCGCCCGGGGACGACCACGTCAAGGCGCACCTATAGTGAGTCGTATTA
**SepCsm cleavage substrates and markers**		
PS364	DNA	5'-GCGGTAATTTTAATGAGATATTTAGAGAACGTATGCCGAAGTATATAAATCATCAGTACAAAGGTAAGAATCACAGTAAACAGCGCGCGG
PS365	DNA	5'-CCGCGCGCTGTTTACTGTGATTCTTACCTTTGTACTGATGATTTATATACTTCGGCATACGTTCTCTAAATATCTCATTAAAATTACCGC
EC primer 1	RNA	5'-GUUUACUGUG
PS365_20	DNA	5'-CCGCGCGCTGTTTACTGTGA
PS365_30	DNA	5'-CCGCGCGCTGTTTACTGTGATTCTTACCTT
PS365_40	DNA	5'-CCGCGCGCTGTTTACTGTGATTCTTACCTTTGTACTGATG
PS365_50	DNA	5'-CCGCGCGCTGTTTACTGTGATTCTTACCTTTGTACTGATGATTTATATAC
PS365_60	DNA	5'-CCGCGCGCTGTTTACTGTGATTCTTACCTTTGTACTGATGATTTATATACTTCGGCATAC
PS365_70	DNA	5'-CCGCGCGCTGTTTACTGTGATTCTTACCTTTGTACTGATGATTTATATACTTCGGCATACGTTCTCTAAA
PS365_80	DNA	5'-CCGCGCGCTGTTTACTGTGATTCTTACCTTTGTACTGATGATTTATATACTTCGGCATACGTTCTCTAAATATCTCATTA
**Substrates used for cryo-EM of RNA-bound TthCsm**		
RNA 4.5	RNA	5'-GGAUAUGCGCCUUGACGUGGUCGUCCCCGGGCGCCUUAUCUACGGUAUCA
ssDNA, NC	DNA	5'-GTTCTTTACGATGCCATTGGGATAGGTCTTTAAAAAGGCCGTAATATCCAGCTGAACGGTCTGGTATCAACGGTGGTATATCCAGTGA

Supplementary Table 2. EM data collection, refinement and validation statistics

	**Csm-TEC complex (Negative staining, EMD-0455)**	**Csm-crRNA-ssRNA complex (Cryo-EM, EMD-0454, PDB-6O1O)**
**Data collection and processing**		
Magnification	49,000	24,500
Voltage (kV)	120	300
Electron exposure (e–/Å^2)	~58	~45
Defocus range (μm)	~0.8-1.5	1.0~3.0
Pixel size (Å)	2.18	1.08
Symmetry imposed	C1	C1
Initial particle images (no.)	100,223	626,621
Final particle images (no.)	8,351	87,321
Map resolution (Å)<br>FSC threshold	17<br>at 0.143	3.8<br>at 0.143
Map resolution range (Å)	14~22	3.5~5.5
**Refinement**		
Initial model used (PDB code)		N/A
Model resolution (Å)<br>FSC threshold		3.93
Model resolution range (Å)		3.5~5.5
Map sharpening *B* factor (Å^2)		-111.3
Model composition<br>Non-hydrogen atoms<br>Protein residues<br>Nucleotides		15193<br>2757<br>66
Overall *B* factors (Å^2)		
R.m.s. deviations<br>Bond lengths (Å)<br>Bond angles (°)		0.007<br>1.343
Validation<br>MolProbity score<br>Clashscore<br>Poor rotamers (%)		2.01<br>8.02<br>1.02%
Ramachandran plot<br>Favored (%)<br>Allowed (%)<br>Disallowed (%)		89.93%<br>9.39%<br>0.68%
